# The Long Reach of Unemployment: Sensitizing or Inoculating Employee Reactions to Job Insecurity?

**DOI:** 10.1007/s10869-025-10052-5

**Published:** 2025-10-07

**Authors:** Maike E. Debus, Tahira M. Probst, Andrea Bazzoli, Hyun Jung Lee

**Affiliations:** 1 Institute of Work and Organizational Psychology, University of Neuchâtel, Rue Emile-Argand 11, 2000 Neuchâtel, Switzerland; 2 Department of Psychology, Washington State University, Vancouver, WA 98686 USA; 3 Department of Psychology, Baruch College & CUNY Graduate Center, 55 Lexington Ave., New York, NY 10010 USA

**Keywords:** Job insecurity, Unemployment experiences, Economic stress, Stress sensitization, Stress inoculation

## Abstract

Adopting a life course perspective, we examined how prior experiences of unemployment might affect reactions to later experiences of job insecurity. In doing so, we applied a strong inference approach and pitted two theoretically derived predictions against each other, namely the stress sensitization and stress inoculation perspectives. According to the stress sensitization perspective, prior experience with unemployment would amplify negative reactions to job insecurity. In contrast, the stress inoculation perspective suggests that individuals with prior unemployment experiences might exhibit attenuated negative reactions to job insecurity. This is because such episodes are appraised as more normative throughout one’s career and are seen as an obstacle that can be successfully overcome. We tested our assumptions using four different archival datasets (total *N* = 1638) from the US and Germany, while considering self-evaluative, affective, attitudinal, performance, and health-related outcomes. Studies 1 to 3 found stronger support for the stress inoculation hypothesis, whereby individuals with prior unemployment experiences reacted less negatively to current job insecurity. In a comparison of explanatory mediating mechanisms in study 4, results indicated that the form of the interaction might depend on what is being triggered by perceived job insecurity (i.e., exacerbated feelings of frustration as reflecting the stress sensitization mechanism vs. attenuated relative deprivation as reflecting the stress inoculation mechanism). By adding a life course perspective to the study of job insecurity, our research broadens the investigation of boundary conditions. We discuss the role of country-level social security and time of data collection to explain the pattern of findings.

Job insecurity—a person’s concern about their future employment stability—is one of the most pertinent economic stressors that employees currently experience (e.g., Shoss, 2017; Sinclair et al., 2024). Underlining the stressfulness of job insecurity, meta-analyses have shown that feelings of job insecurity are related to a host of detrimental outcomes, such as more negative job attitudes, worse health, and lower work motivation and performance (e.g., Jiang & Lavaysse, 2018). As such, it is not surprising that large-scale opinion surveys consistently reveal that job *security* is one of the top things that employees and job seekers alike want from their jobs (e.g., Talentlms, 2024; Wigert, 2022). Because job insecurity per se is hard to change, researchers have spent a great deal of attention on examining boundary conditions that moderate the job insecurity–outcome link in an effort to identify who suffers more vs. less from job insecurity (e.g., Cheng & Chan, 2008; Lee et al., 2018).

Thus far, a vast array of moderators at the individual, organizational, and societal levels have been identified (see Jiang, 2025, for an extensive overview). At the individual level, for example, moderators related to individual traits (e.g., neuroticism, self-efficacy), intrapersonal states (e.g., type of employment contract, perceived employability), and coping behaviors (e.g., cognitive reappraisal, self-care strategies) have been shown to moderate the relationship between job insecurity and outcomes. At the organizational level, factors related to how organizations and its representatives treat their employees (e.g., organizational justice, leader-member exchange) as well as job design features (e.g., autonomy) affect how employees appraise and cope with job insecurity. Finally, at the societal level, moderators related to cultural values (e.g., uncertainty avoidance, individualism), economic conditions (e.g., economic downturns, unemployment rate), and governmental policies (e.g., safety net policies, generosity of unemployment benefits) have been identified. Notably, most of these boundary conditions appear to be contemporaneous, that is, they refer to person- or situational factors happening at the same point in time.

Yet, people do not live in a temporal vacuum; rather, we all have a history and a set of past experiences that shape who we are today and that can color our interpretation of and reactions to presently experienced events (e.g., Debus & Unger, 2024; Thoits, 2010). Taking these earlier experiences into consideration is crucial to achieving a more nuanced understanding of job insecurity and its outcomes and to view job insecurity and economic stress more broadly as a whole-life experience. In the present study, we thus adopt a life course perspective (e.g., Elder & Shanahan, 2006; Zacher & Froidevaux, 2021) to examine the following research question: How do *prior unemployment spells* affect employee reactions to *current threats of job loss (i.e., perceived job insecurity)*? This is a particularly apt question, since employment provides numerous valuable latent and manifest benefits, including time structure, a sense of collective purpose, opportunities for social contact, social status, and activity (Jahoda, 1981). We argue that individuals who have prior experience with a lack of employment might react differently to the potential loss of their employment compared to those who have never weathered that situation. As such, by considering a person’s prior unemployment history as a boundary condition we explicitly take a retrospective view, thus extending the present focus on contemporaneous moderators of the job insecurity-outcome link.

As Paul et al. (2023) note in their recent meta-analysis of employment status, individuals who are not employed may be in this state *voluntarily* (i.e., out of the labor force due to preferences for homemaking or the pursuit of educational or leisure activities) or *involuntarily* (i.e., being out of the labor force but desiring to return). Interestingly, whereas involuntary unemployment was associated (as expected) with across-the-board losses of the latent and manifest benefits of employment, even individuals who were voluntarily unemployed exhibited significant resource losses—most notably in the areas of time structure, collective purpose, and status—compared to employed individuals (Paul et al., 2023). Based on these findings, the authors concluded that it is the state of employment that is the main provider of the latent functions. Thus, in our current study, the reason for these prior unemployment spells is less important than the fact of their occurrence, as we argue that it is an individual’s prior experience with the state of unemployment that may color their reactions to currently perceived job insecurity.

We focus on these earlier experiences of unemployment spells for two reasons. First, unemployment reflects what is anticipated when individuals experience job insecurity—namely being in a situation without a job (e.g., Probst, 2018; Shoss, 2017). Due to the strong conceptual link between both constructs, theoretical reasoning would suggest that earlier unemployment experiences affect how individuals appraise and interpret current job insecurity. Second, although unemployment is a critical life event that has been associated with numerous adverse short- and long-term outcomes (e.g., worse mental and physical health, increased odds of suicide ideation, sucide attempts, and mortality, Amiri, 2022; McKee-Ryan et al., 2005; Paul & Moser, 2009; Wanberg, 2012), individuals may also use periods of unemployment to potentially develop coping skills to increase their resilience (e.g., to develop mechanisms of time structure, social support and other latent benefits of employment even while in a state of unemployment, Paul et al., 2023) and for reflecting about themselves and their careers (Zikic & Klehe, 2006). How earlier spells of unemployment may affect reactions to current job insecurity is, to date, not very clear, which is why we particularly focus on the interplay between job insecurity and unemployment as two major economic stressors.

To answer our proposed research question, we apply a strong inference approach (e.g., Platt, 1964) and pit two foundational theories of stress—conservation of resources theory (COR, Hobfoll, 1989, 2001; Hobfoll et al., 2018) and the transactional theory of stress and coping (TTSC, Lazarus, 1999, 2001) — against each other. Whereas both theories suggest detrimental *main effects* of job insecurity on cognitive, behavioral, and strain outcomes, these theories suggest opposing predictions in terms of the long-lasting “after-effects” of unemployment experiences on employee reactions to job insecurity. We thus contrast these theories to develop competing perspectives and predictions regarding how prior unemployment may *moderate* job insecurity–outcome relationships. Derived from COR theory, the *stress sensitization perspective* suggests that “adverse experiences sensitize individuals to heightened sensitivity and reactivity to subsequent stress” (Luo et al., 2021, p. 2). Previous stress experiences would make individuals more vulnerable to recent and future stress experiences, potentially due to loss spirals occurring as a result of resource loss from those earlier events (Hobfoll, 1989; see also Thoits, 2010). Following the stress sensitization perspective, individuals would react more negatively to current job insecurity if they have experienced unemployment in the past. In contrast, the *stress inoculation perspective* (Meichenbaum & Deffenbacher, 1988) derived from TTSC proposes that prior exposure to stressful life events changes stressor appraisal and builds our repertoire and reservoir of coping mechanisms. This can then lead to greater resilience and can blunt the negative impact of subsequently encountered stressors. Thus, much like a vaccine trains our body to fight off future pathogens we encounter, the experience of past challenging events such as episodes of unemployment might equip individuals to better cope with a subsequently experienced threat of job loss (i.e., job insecurity). To identify potential patterns and to examine the robustness and generalizability of these proposed competing interactive effects, we consider a variety of outcomes in the self-evaluative, attitudinal, affective, performance, and health domains that have all been well-established in the job insecurity literature (e.g., Jiang & Lavaysse, 2018; Lee et al., 2018; Sverke et al., 2002).

Following our development of these competing perspectives, we extend our reasoning on the interactive effect of job insecurity and unemployment to consider the role of two potential mediators (i.e., frustration and relative deprivation) and use this refined moderated mediation model to further explore its effect on several individual outcomes. On the one hand, people who have experienced unemployment in the past and are presently at risk of again losing their job represent a potentially very vulnerable group (e.g., Lucas et al., 2004) and therefore may be more frustrated by the repeated exposure to this economic stressor. On the other hand, individuals with prior unemployment episodes may consider it more normative for careers to be nonlinear with disruptions, and may also have developed coping skills because of these experiences (Danckert, 2017). As such, they may exhibit attenuated relative deprivation when experiencing job insecurity. Accordingly, it is important to investigate the mechanisms that explain any observed interactive effects in order to develop future interventions. In doing so, we can more precisely map the underlying theoretical processes derived from the stress sensitization vs. stress inoculation perspectives, allowing us to gain a deeper understanding of the involved phenomena and their related mechanisms.

Our paper is organized as follows: we begin by briefly reviewing extant theories and empirical evidence regarding the work-related effects of job insecurity before considering in greater detail the theoretical foundation for the competing stress sensitization vs. stress inoculation propositions, as well as potential mediating mechanisms. Finally, with an eye toward constructive replication (Köhler & Cortina, 2021), we map out a series of cross-sectional and multi-wave studies using different operationalizations of job insecurity and prior unemployment spells to test these theoretical propositions and potential explanatory mechanisms. Figure 1 displays the conceptual model of our research, and Table 1 provides an overview of the datasets used to test specific hypotheses.

**Fig. 1:**
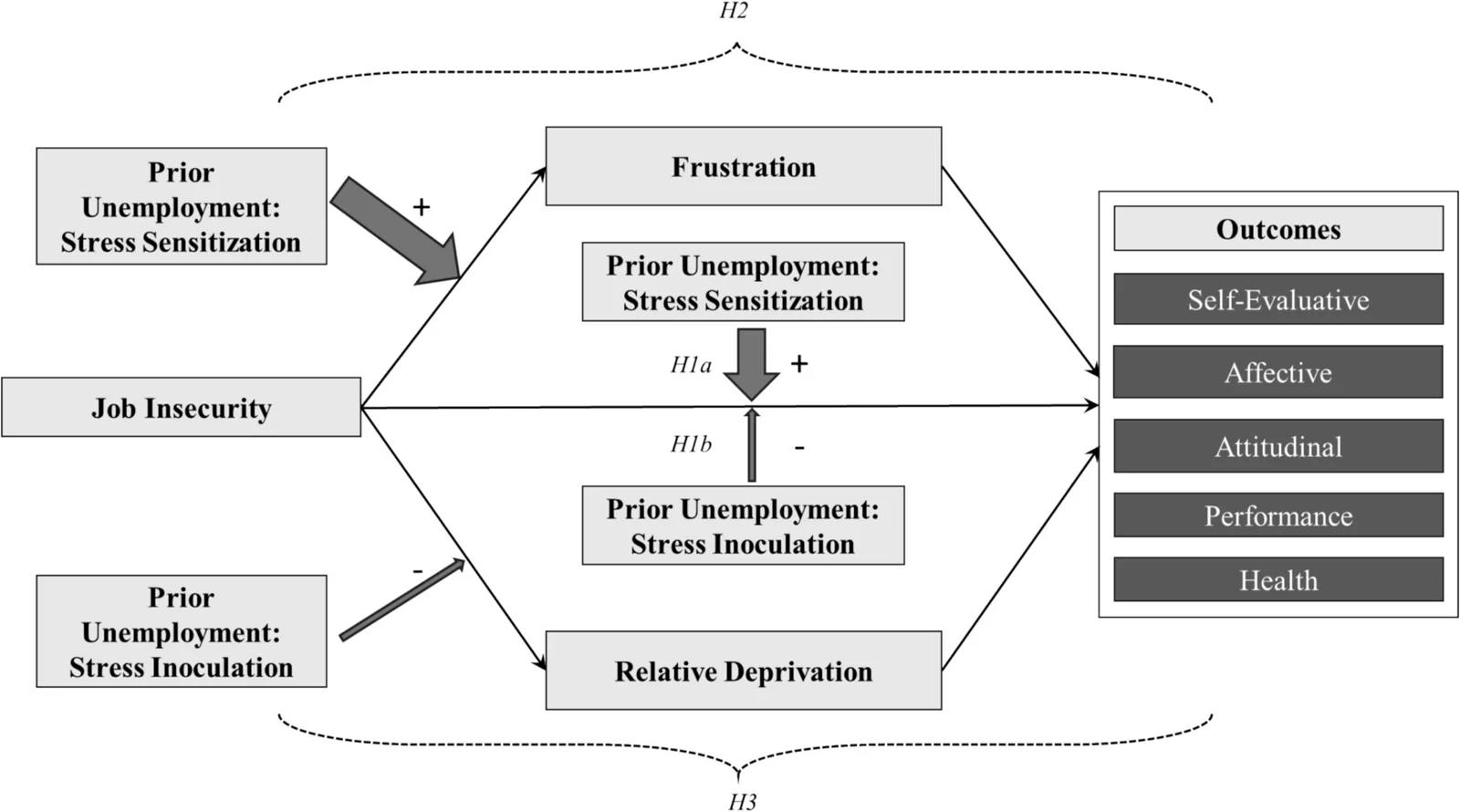
Conceptual model. H, hypothesis

**Table 1:** Hypotheses and studies

Hypothesis		Study 1	Study 2	Study 3	Study 4
1a	Stress sensitization hypothesis (moderation)^a^	X	X	X	
1b	Stress inoculation hypothesis (moderation)^a^	X	X	X	
2	Frustration as the underlying mechanism of the stress sensitization effect (moderated mediation model)^b^				X
3	Relative deprivation as the underlying mechanism of the stress inoculation effect (moderated mediation model)^b^				X

^a^Hypotheses 1a and 1b were tested on all the outcomes in studies 1 to 3

^b^Outcomes in study 4 to test the moderated mediation were self-evaluative outcomes (demands-abilities fit, needs-supplies fit), attitudinal outcomes (intention to remain, job satisfaction), and performance outcomes (task performance, incivility)

## Outcomes of Job Insecurity

Predictions regarding the outcomes of job insecurity are typically drawn from stress theories (e.g., Jiang & Lavaysse, 2018; Lee et al., 2018; Sverke et al., 2002), with COR theory (Hobfoll, 1989, 2001; Hobfoll et al., 2018) and TTSC (Lazarus, 1999, 2001) being most often used (e.g., Bazzoli & Probst, 2022; Shoss, 2017; Sinclair et al., 2024). Both theories view job insecurity as a stressor that results in negative outcomes. First, according to COR theory, job insecurity represents a threat to the valued resource of employment, as well as the related resources of identity, income, social connection, and social status (e.g., Selenko & Batinic, 2013; Shoss, 2017). If individuals experience such a resource threat, they experience strain and try to protect their remaining resources by withdrawing from any activities that require them to invest effort. Second, TTSC explains individuals’ responses to job insecurity as an interactive process between an individual and their environment. The theory predicts that when individuals encounter job insecurity, they likely appraise this situation as an uncontrollable threat with limited coping options. Due to the uncontrollability of this situation, individuals are expected to experience aversive affective states, along with lower motivation, performance, and worse health (e.g., Shoss, 2017; Sinclair et al., 2024). In essence, both COR theory and TTSC highlight the threatening and uncontrollable nature of job insecurity that puts both manifest and latent resources at stake (e.g., Jahoda, 1981). As a consequence, appraising the situation of job insecurity makes individuals evaluate their work situation and themselves more negatively, develop more negative affective reactions and more negative attitudes about their job situation, decrease their performance and increase counterproductive behaviors as a means to retaliate against the organization, and show worse health outcomes due to constant worry and rumination.

Empirically, a vast literature (see Cheng & Chan, 2008; Jiang & Lavaysse, 2018; Sverke et al., 2002, 2019, for meta-analyses) supports these theories, finding that job insecurity affects employee outcomes in five key domains: *self-evaluative*, *affective*, *attitudinal*, *performance*, and *health-related outcomes*. First, in terms of *self-evaluation outcomes*, job insecurity has been found to be negatively associated with *perceived demands-abilities fit* (i.e., a person’s perceived compatibility between their formal job requirements/job tasks and their knowledge, skills, and abilities (KSAs, Edwards, 1991; see Lu et al., 2014)), *perceived needs-supplies fit* (i.e., the degree to which individuals perceive that the job fulfills their goals, values, and desires, Edwards, 1991; see Lu et al., 2014), and *task-related self-efficacy* (i.e., individuals’ confidence of possessing what is needed to master their work tasks, Bandura, 1997; Barbaranelli et al., 2018; see Guarnaccia et al., 2018; Van Hootegem et al., 2021). Second, in terms of *affective outcomes*, job insecurity has been associated with more *negative affect* and reduced *job-related affective well-being*. Third, job insecurity has been shown to be associated with adverse *attitudinal outcomes*: lower *job satisfaction*, higher *turnover intentions*, and lower *career commitment* (i.e., a person’s attitude toward their work or field, Blau, 1985; for initial evidence see Goulet & Singh, 2002). Fourth, in terms of *performance outcomes*, job insecurity results in lower *task performance* as in-role performance of the individual’s core job description, higher *incivility* as low-intensity, rude, or disrespectful behaviors that violate norms of mutual respect in the workplace, lower *safety compliance* as employees’ adherence to established safety rules, regulations, and procedures in the workplace, and higher *cognitive failures* as lapses in attention, memory, or action that result in errors or mistakes during tasks (e.g., Probst et al., 2020a, 2020b). Fifth, in terms of *health outcomes*, job insecurity has been shown to be associated with lower *mental health*, lower *physical health*, and more *sleep disturbances* as disruptions in the normal pattern, quality, or quantity of sleep (e.g., Choi et al., 2020). We selected these five key domains because they have been repeatedly established as critical outcomes of job insecurity in earlier research, thus allowing us to examine the robustness and generalizability of the proposed interaction effects, as well as to highlight the extent to which support for the stress sensitization vs. stress inoculation perspectives emerges across these different classes of outcomes.

## The Interactive Effect Between Job Insecurity and Unemployment Experiences

While there is ample theoretical and empirical evidence for the abovementioned main effects of job insecurity, meta-analytic findings strongly indicate the presence of potential moderating effects given heterogeneity in observed effect sizes (e.g., Cheng & Chan, 2008; Sverke et al., 2002). Specifically, we posit that prior unemployment spells will moderate employee reactions to subsequently experienced job insecurity (see also Smid et al., 2012). While both COR theory (Hobfoll, 1989, 2001) and the TTSC (Lazarus, 1991, 1999) propose detrimental main effects of job insecurity on various outcomes (as explained above), these theories suggest two potentially opposing effects—a *stress sensitization* effect (Hobfoll, 1989, 2001) versus a *stress inoculation* effect (Lazarus, 1991, 1999)—by which those previous unemployment experiences may affect an individual’s reaction to later job insecurity. Below, we discuss the theoretical foundation and empirical support for each perspective and delineate how each would predict opposing moderating effects.

### The Stress Sensitization Perspective

According to COR theory (Hobfoll, 1989, 2001), individuals with fewer resources are more vulnerable to subsequent resource loss or threats. Stress sensitization builds on this principle, describing heightened stress responsiveness due to prior stressor exposure (Luo et al., 2021). Specifically, a prior stressor, as a temporally preceding moderator, intensifies the impact of current stressors on distress. This heightened sensitivity occurs because individuals with lower resource pools lack the “buffer” needed to absorb further threats and losses, leaving them more vulnerable to stressors (Hobfoll, 1989, 2001). In the context of unemployment, this perspective suggests that individuals who have experienced unemployment will have fewer resources available compared to those who have not, even if they are re-employed (for a similar reasoning in a different context see van Woerkom et al., 2016). The lower resource pool will make individuals with prior unemployment experiences more vulnerable to job insecurity such that job insecurity–outcome relationships would be exacerbated.

Providing evidence for how resource pools diminish during unemployment, research has shown that unemployment is accompanied by the loss of several resources, such as decreased self-esteem (Goldsmith et al., 1997), decreased locus of control (Goldsmith et al., 1996), a decreased feeling of collective purpose, decreased financial resources, fewer social contacts and activity, and a disrupted time structure (e.g., Atkinson et al., 1986; Jahoda, 1982; Selenko et al., 2011). While some resources, such as time structure and daily activity, may be restored with re-employment, others, like social networks and self-esteem, are more difficult to rebuild. For example, unemployed individuals tend to have fewer social contacts both at work and outside of work, which can negatively impact their sense of prestige and self-esteem (e.g., Selenko et al., 2011; Voss et al., 1999). Additionally, they report lower satisfaction with their social lives (Powdthavee, 2012), potentially due to withdrawal from social activities due to financial reasons, but also due to diminished self-esteem (e.g., Kinnunen et al., 2003). Even when individuals regain employment, establishing new social contacts with work colleagues and/or re-establishing private social contacts requires significant time and effort (e.g., Hall, 2019). Furthermore, unemployment often carries stigma (e.g., Garcia-Lorenzo et al., 2021), which may threaten individuals’ self-esteem and confidence on a long-term basis and which likely carries after-effects even after having regained employment.

Moreover, although re-employment is generally viewed as a positive outcome, it does not always restore prior levels of resources. Many individuals find themselves in jobs with poorer job characteristics, such as lower salaries, part-time work, and reduced job authority or occupational status compared to their previous job (e.g., Brand, 2006; Vesalainen & Vuori, 1999; Waters, 2007). These experiences can leave lasting “scars” on individuals’ resource pools. Scarring refers to the long-term consequences of unemployment that may persist even after re-employment (Clark et al., 2001). For instance, individuals may internalize unemployment as part of their self-concept, which can further erode self-esteem and resilience to future stressors. Taken together, these aspects and experiences likely damage individuals’ resource pools on a long-term basis, even after having regained employment.

Longitudinal studies provide indirect evidence of the enduring impact of unemployment on individuals’ resource pools. For example, Lucas et al. (2004) demonstrated that life satisfaction significantly decreases when individuals experience a spell of unemployment. When regaining employment, life satisfaction increases again, yet does not reach individuals’ original baseline level of life satisfaction (see also Clark et al., 2001). Similarly, Luhmann and Eid (2009) found that life satisfaction further decreases with each new spell of unemployment. Individuals react more negatively to each spell of unemployment, thus likewise indicating a sensitization pattern. In sum, the stress sensitization perspective suggests that prior unemployment contributes to long-term lowered resource pools among individuals that (partly) persist even after regaining employment. Due to these diminished resources (e.g., lower self-esteem, less dense social networks, lower financial resources), individuals with prior unemployment are less well equipped to cope with the threat of job loss (i.e., job insecurity). As a result, individuals with prior unemployment would be more sensitive or vulnerable to job insecurity as a resource threat compared to employees without prior unemployment. We thus predict:

- *Hypothesis 1a*: Prior unemployment exacerbates the relationship between job insecurity and its outcomes.

### The Stress Inoculation Perspective

In contrast, Lazarus’s TTSC provides a valuable framework for understanding the stress inoculation perspective (Meichenbaum & Deffenbacher, 1988) which posits that prior exposure to stressors allows individuals to develop a broader pool of cognitive, affective, and behavioral coping resources that can be drawn upon when managing future stressors. In highlighting the dynamic interplay between the individual and the environment, stress and coping are expected to be determined not only by exposure to an objective stressor but also by the individual’s primary appraisal of its significance (i.e., the extent to which they perceive the potential stressor as a threat) and their secondary appraisal of their capacity to manage (i.e., cope with) the potential stressor.

From this perspective, an individual’s past experience of unemployment might influence the way they perceive and react to job insecurity, as they might align their expectations with the notion that periods of unemployment are not uncommon. In other words, individuals who have navigated prior periods of unemployment may recalibrate their primary appraisals of job insecurity (e.g., by perceiving it as a more normative and expected aspect of employment rather than as a significant threat or by reframing it as an opportunity for career exploration, personal growth, or skill development, Zikic & Klehe, 2006). Through such cognitive reframing, prior exposure to unemployment may lead individuals to appraise the potential stressor of job insecurity as less of a hindrance or significant threat to one’s career,^1^ and in turn, attenuate the stress response.

Additionally, TTSC would imply that a *lack* of exposure to a given stressor (such as unemployment) may limit the development of valuable coping resources in terms of secondary appraisal, leaving individuals without that prior exposure less equipped to manage stressors such as a presently perceived threat to one’s employment (i.e., job insecurity). For example, individuals who have experienced unemployment in the past and subsequently gained employment might appraise themselves as a person who knows how to enhance their external employability by developing skills and strategies that can be applied on the job market should they lose their current job. As a result, current job insecurity may ultimately be perceived as less of a threat to these individuals compared to employees who have never before been unemployed and might not have had an opportunity to build those skills or resources.

In sum, drawing upon this model, the stress inoculation perspective would hypothesize that individuals who have previously navigated periods of unemployment may develop a cognitive framework or mindset that incorporates job insecurity as a more normative aspect of the employment trajectory (leading to attenuated primary appraisals of threat) as well as a more robust repertoire of skills to cope with a perceived threat to one’s employment (leading to more positive secondary appraisals of coping with the potential stressor). Together, this would predict mitigated negative self-evaluative, affective, attitudinal, performance, and health responses associated with uncertainty about one’s job.

While research examining potential stress inoculation effects is limited in organizational contexts, there is empirical evidence for the underlying theoretical processes. For example, Seery and colleagues found that individuals with moderate prior exposure to stressful life events showed less physical impairment and lower healthcare utilization in response to chronic back pain (Seery et al., 2010) and also displayed fewer negative responses to acute stressors such as induced pain (via a cold pressor task) and a high-pressure cognitive test (Seery et al., 2013). Kok et al. (2021) found that while cumulative exposure to negative life events (e.g., death of a family member, involuntary unemployment, divorce) was associated with more depressive symptoms, individuals with more prior negative life events exhibited attenuated responses to more recently experienced stressors, suggesting that repeated exposure had built resilience. Unfortunately, while Kok et al. (2021) included unemployment as a negative life event, they did not separately evaluate the unique inoculating effect due to experiencing this specific stressor. Similarly, a recent study by Klopack et al. (2022) found that prior experienced financial strain attenuated responses to subsequent life stressors, including unemployment and demotion at work. Together, this is suggestive that the stress inoculating effects observed in other domains might also translate to exposure to economic stressors, such as unemployment and job insecurity.

Based on our theoretical foundation and the available empirical evidence, we predict that:

- *Hypothesis 1b*: Prior unemployment attenuates the relationship between job insecurity and its outcomes.

## Extending the Interactive Effect of Job Insecurity and Unemployment Experiences to Mediating Mechanisms: A Case of Moderated Mediation

Building upon our earlier propositions from the stress sensitization and stress inoculation perspectives, we also seek to more explicitly integrate and test potential underlying mechanisms. First, drawing on the stress sensitization perspective and stress-related mediating processes, we suggest that job insecurity is indirectly related to the outcomes via *frustration*, and we anticipate this indirect effect to be more pronounced among employees with prior experiences of unemployment, thus resulting in a moderated mediation effect (e.g., Shoss, 2017; Vander Elst et al., 2012). Second, we suggest that job insecurity is also indirectly related to the outcomes via fostering a sense of *relative deprivation* (i.e., a negative comparison of one’s current situation relative to similar others or oneself at an earlier point, Crosby, 1984; Smith et al., 2011). However, reflecting the stress inoculation perspective, we anticipate this indirect effect to be less pronounced among employees with prior experiences of unemployment, thus likewise resulting in a moderated mediation effect. Below we expand upon our rationale for these proposed effects.

### Frustration to Explain the Stress Sensitization Effect

Hobfoll et al. (2018) cautioned against the assumption that resources are static, but rather argued for the notion that resources can be cultivated or, alternately, frustrated. As such, one of the central mechanisms underlying the effects of resource loss, threat, and vulnerability in line with stress sensitization is resource frustration (Hobfoll et al., 2018). Frustration by itself is a discrete emotion that occurs when individuals feel they do not have enough resources to achieve their goals (Eissa & Lester, 2017; Fox & Spector, 1999; Reio, 2011) and/or if certain conditions block or interfere with their goals (e.g., Spector, 1978). This reasoning further implies that job insecurity would be indirectly related to further negative outcomes via the experience of frustration, precisely because job insecurity interferes with individuals’ goals of cultivating stable employment.

Indeed, early work on the experience of frustration has identified job insecurity as a primary source of this emotion (in addition to the frustrating nature of the work itself, a lack of promotion opportunity, role ambiguity, organizational change, and physical isolation from the community, Eaton, 1952; see also Spector, 1978). Relatedly, researchers have highlighted that job insecurity as the prospect of losing one’s employment “means the frustration of some fundamental human needs,” such as the needs for survival, relatedness, and self‐determination (De Witte et al., 2016, p. 19). Based on this reasoning, we argue that job insecurity indirectly leads to the aforementioned outcomes via frustration, that is, frustration mediates the relationships between job insecurity and the aforementioned outcomes. Next, building upon this mediating effect and based on COR theory’s notion of increased vulnerability due to already lost or threatened resources, we postulate that prior unemployment strengthens the relationship between job insecurity and frustration (i.e., the a-path of the mediation chain), resulting in a first-stage moderated mediation effect. Based on our earlier arguments, job insecure individuals are likely to be even more frustrated by their situation if they have experienced prior unemployment as this experience can leave lasting damage to their resource pools, even after they have regained employment.

- *Hypothesis 2*: Prior unemployment moderates the indirect effect of job insecurity on outcomes via frustration, such that the indirect effect is stronger under higher levels of prior unemployment.

### Relative Deprivation to Explain the Stress Inoculation Effect

Individuals do not respond to conditions solely based on their objective quality, but rather based on cognitive appraisals entailing interpersonal comparisons. When individuals perceive their conditions as worse off compared to relevant others or to their own past experiences, they are likely to experience relative deprivation, a psychological state characterized by feelings of unfairness and angry resentment (Crosby, 1976; Smith et al., 2011). This reasoning aligns with TTSC, which emphasizes the role of cognitive appraisal in stress reactions and highlights emotional responses, such as anger, when individuals perceive threats to their dignity (Lazarus, 1999). Within the present study, job insecure individuals are likely to engage in upward social comparisons. They may compare themselves to peers with similar qualifications who appear more secure in their jobs, or to their own past (more favorable) employment situation. This comparison can give rise to feelings of being unfairly disadvantaged, because job insecurity is seen as violating psychological contracts or norms of reciprocity between employee and employer (e.g., Reisel et al., 2010; Rousseau, 1995). Thus, job insecurity is likely to evoke relative deprivation, serving as a mediating mechanism that links job insecurity to its outcomes. Indeed, research has shown that job insecurity is associated with deprivation-related feelings (for feelings of injustice see Lazauskaite-Zabielske et al., 2019; López Bohle et al., 2021; Piccoli & De Witte, 2015; for feelings of anger see Reisel et al., 2010). Moreover, relative deprivation has been shown to be related to numerous detrimental outcomes (e.g., Erdogan et al., 2018; Mishra & Novakowski, 2016; Wickham et al., 2014).

Next, building upon this mediating effect and recalling our theorizing around the stress inoculation effect embedded in TTSC (Lazarus, 1991, 1999), we hypothesize that prior unemployment attenuates the relationship between job insecurity and relative deprivation (i.e., the a-path of the mediation chain), resulting in a first-stage moderated mediation effect. As mentioned before, individuals who have previously experienced unemployment may recalibrate their primary appraisals, perceiving job insecurity as a more normative and less threatening part of their employment trajectory. This shift reduces the perceived discrepancy between their own situation and that of similar others, thereby weakening the experience of relative deprivation. At the same time, such individuals may also develop more positive secondary appraisals, as their past experience with unemployment may have equipped them with a larger repertoire of coping skills (Patton & Donohue, 1998; Wanberg, 1997). This enhanced coping capacity may result in their perception that they may be better off than similar others in response to job insecurity, as well as still being better off than having been unemployed before. Together, these appraisal shifts may reduce the emotional intensity of upward comparisons and thus attenuate the link between job insecurity and relative deprivation.

- *Hypothesis 3*: Prior unemployment moderates the indirect effect of job insecurity on outcomes via relative deprivation, such that the indirect effect is weaker under higher levels of prior unemployment.

## Overview of Studies

We tested our assumptions using four previously collected archival datasets, including one cross-sectional and three multi-wave datasets from the US and Germany. All four datasets stemmed from previous projects led independently by the first and second authors between 2017 and 2021 and were used retrospectively to test our hypotheses. Data analyzed in study 1 were collected by master level students as part of a university research seminar in Germany; data presented in studies 2 and 3 stem from larger projects on economic stressors and workplace safety collected in the US, and study 4 data were from a larger project on economic stressors conducted with German participants. Portions of the data used in this manuscript (analyzed in studies 2, 3, and 4) have been published elsewhere (Bettac & Probst, 2021; Bettac et al., 2021; Debus et al., 2023; Lee et al., 2024; Probst et al., 2020a, c; Petitta et al., 2019, 2020, 2021).

While reliance on archival data limited our ability to standardize operational definitions, measures, and methods across the four studies, these same limitations allowed us to evaluate the extent to which our hypothesized effects were robust due to these varied operationalizations of job insecurity, previous unemployment spells, and outcome variables. As Köhler and Cortina (2021) note, constructive replication is a valuable methodological approach precisely *because* studies test the same hypotheses but with variations in methods, settings, populations, and measurements. By conducting multiple tests, one can strengthen the validity and generalizability of findings by reducing the impact of methodological biases associated with one approach and avoiding capitalization on chance findings.

Our data, research materials, including detailed descriptions of each measure, and analysis code are available at https://osf.io/gqvyc/. The data for the current studies were all analyzed using *Mplus* 8 (Muthén & Muthén, 1998–2017).

## Study 1: Cross-Sectional Dataset from Germany (*N* = 205)

### Study 1 Method

#### Participants

Our first study used cross-sectional data collected from 205 German workers in 2020 via a snowball sampling approach. Students enrolled in a research seminar collected data from their professional networks and by posting study information material on several social networks. This approach is advantageous as it yields a more heterogeneous sample and it enhances the generalizability of findings (Demerouti & Rispens, 2014). In return for their participation, employees received a summary of the findings. Inclusion criteria were (a) providing consent to participate, (b) currently working for pay, (c) being at least 18 years old, (d) not being self-employed, a student, trainee, and/or doing an internship, (e) not having resigned or been dismissed from their current job, and (f) having worked during the preceding four weeks. A majority of participants identified as female (58%). Participants were between 18 and 65 years of age (*M* = 35, *SD* = 13), and worked 40 h per week on average (*SD* = 30) in a variety of industries (e.g., communication; oil, gas, and energy; health care).

#### Measures

*Cognitive job insecurity* was measured using Borg and Elizur’s (1992) 4-item scale, and *previous unemployment* was measured using a binary variable (0 = no previous unemployment spells and 1 = at least one previous unemployment spell). We assessed *job satisfaction* (Bowling & Hammond, 2008, 3-item scale), *turnover intention* (Staufenbiel & König, 2010, 2-item scale), *career commitment* (adapted from Staufenbiel & König, 2010, 2-item scale), and *negative affect* (Segura & González-Romá, 2003, ranging from 1= *not at all* to 5 = *very much*, 3-item scale). Unless otherwise noted, all items used a 7-point rating scale (1 = *strongly disagree* to 7 = *strongly agree*). Items can be seen in our OSF folder.

### Study 1 Results

Means, standard deviations, correlations, and reliability coefficients of study variables are reported in Table S1 (S = supplementary material in the OSF folder). Corroborating earlier research, job insecurity was significantly related to the attitudinal outcomes (i.e., negative relationship with job satisfaction, *r* = − 0.37, *p* < 0.001; positive relationship with turnover intentions, *r* = 0.44, *p* < 0.001; negative relationship with career commitment, *r* = − 0.27, *p* < 0.001) and the affective outcome (positive relationship with negative affect, *r* = 0.15, *p* < 0.05). Before testing our structural hypotheses, we conducted confirmatory factor analyses (CFAs) to ensure that the proposed measurement model fit the data adequately and compared it with alternative measurement models. Results suggested that the proposed five-factor measurement model in which each construct (i.e., cognitive job insecurity, job satisfaction, turnover intention, career commitment, negative affect) was represented by its own latent factor showed close fit to the data [*χ*^2^(67) = 154.90, CFI = 0.95; RMSEA = 0.08; SRMR = 0.05] and fit the data better than an alternative model. Model comparison results can be found in Table S3.

**Fig. 2:**
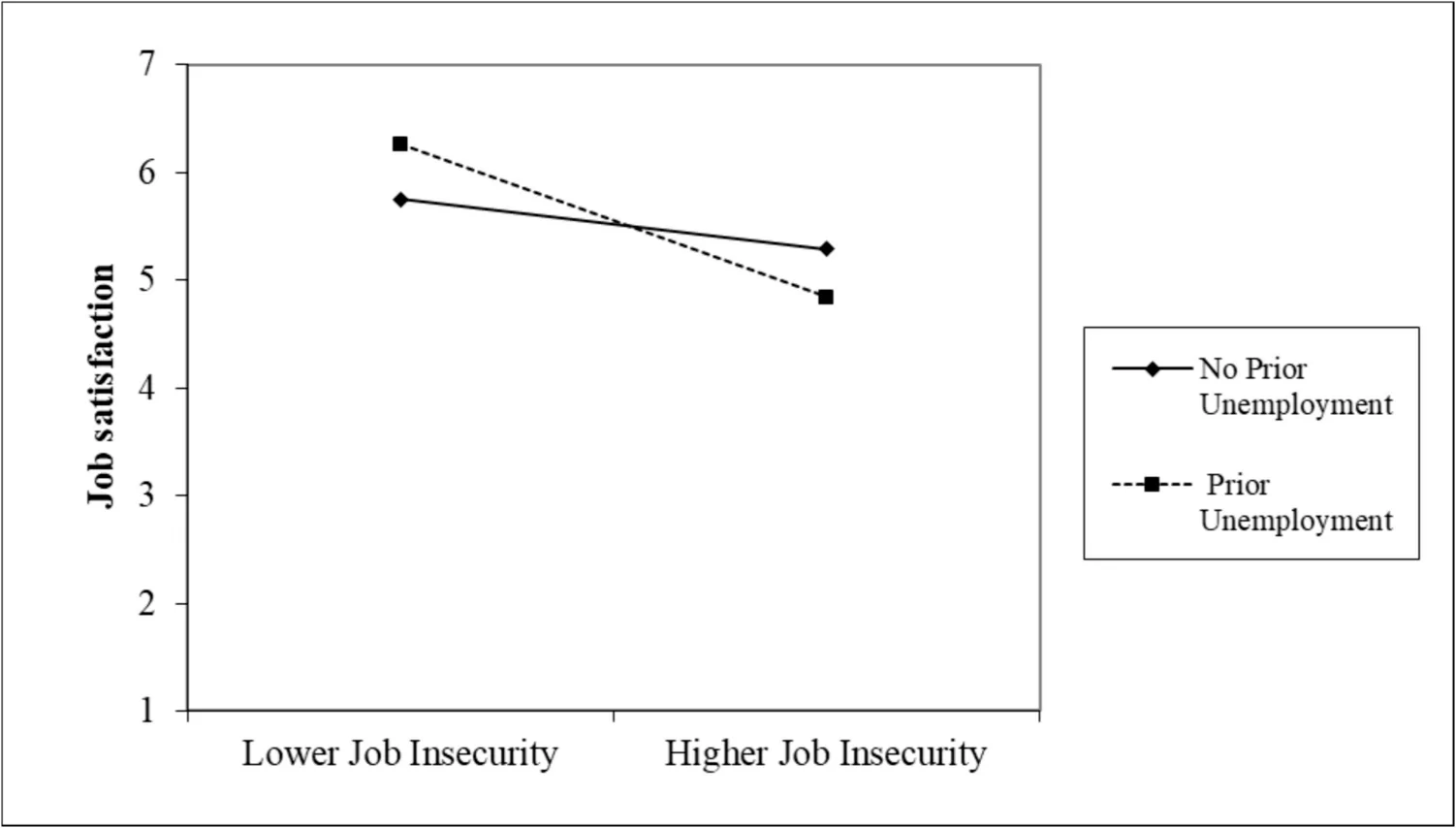
Prior unemployment moderates the job insecurity–job satisfaction relationship (study 1). Lower and higher refer to + 1 and − 1 SD

Before estimating our structural relationships, job insecurity was mean-centered (Cohen et al., 2003), and the product of centered job insecurity and prior unemployment (0 = no, 1 = yes) was used as the interaction term. Parameter estimates, standard errors, and *p*-values are reported in Table 2. Concerning the postulated moderation effects, we found significant job insecurity × prior unemployment interaction terms for the three attitudinal outcomes: job satisfaction (interaction *γ* = − 0.44, *p* = 0.005), turnover intention (interaction *γ* = 0.44, *p* = 0.041), and career commitment (interaction *γ* = − 0.44, *p* = 0.043). Simple slope tests show that employees who had previously experienced unemployment reacted more negatively to current job insecurity; that is, job insecurity was more strongly related to job satisfaction, turnover intention, and career commitment for those who had prior unemployment spells (*γ* = − 0.70, *p* < 0.001; *γ* = 0.93, *p* < 0.001; *γ* = − 0.71, *p* < 0.001, respectively) than those who had no unemployment spells (*γ* = − 0.26, *p* = 0.001; *γ* = 0.49, *p* < 0.001; *γ* = − 0.26, *p* = 0 0.003, respectively). The moderation patterns are graphically depicted in Figs. 2, S1, and S2. These results suggest that employees who had previously experienced unemployment reacted more negatively to current job insecurity, providing support for the stress sensitization perspective (Hypothesis 1a).

**Table 2:** Results from study 1

Outcome		Parameter estimate	*SE*	*p*
*Attitudinal outcomes*	*Career commitment*			
	Intercept	4.77	0.25	< 0.001
	Prior UE	0.42	0.28	0.137
	Job insecurity	− 0.26	0.09	0.003
	UE × job insecurity	− 0.44	0.22	0.043
	* R*^*2*^	0.10		
	*Job satisfaction*			
	Intercept	5.53	0.09	< 0.001
	Prior UE	0.04	0.20	0.843
	Job insecurity	− 0.26	0.06	< 0.001
	UE × job insecurity	− 0.44	0.16	0.005
	* R*^*2*^	0.17		
	*Turnover intention*			
	Intercept	2.74	0.12	< 0.001
	Prior UE	− 0.16	0.28	0.557
	Job insecurity	0.49	0.09	< 0.001
	UE × job insecurity	0.44	0.21	0.041
	* R*^*2*^	0.21		
*Affective outcome*	*Negative affect*			
	Intercept	2.37	0.07	< 0.001
	Prior UE	0.22	0.16	0.156
	Job insecurity	0.06	0.05	0.252
	UE × job insecurity	0.23	0.12	0.055
	* R*^*2*^	0.05		

*N* = 205. Parameter estimates are unstandardized

*SE* standard error, *UE* unemployment experiences (0 = no and 1 = yes)

## Study 2: 2-Wave Dataset in USA (*N* = 390)

Building on study 1 results, we next extended our analysis to another country where we also considered a broader set of outcomes and used a different operationalization of job insecurity. We also used a two-wave dataset to address concerns related to the cross-sectional nature of the data used in Study 1.

### Study 2 Method

#### Participants

We collected this dataset (*N* = 390) via Amazon Mechanical Turk (MTurk) at two time points with a 1-month lag in 2017. Participants were compensated $4 for completion of the first survey and $5 for the follow-up survey. Using pre-screening qualifications recommended by Peer et al. (2014), we only invited MTurkers who had (a) a job outside the MTurk platform, (b) a minimum of 90% prior approval rating for completed tasks, and (c) prior successful completion of at least 100 tasks. The sample was predominantly male (56%). The average age of respondents was 35.71 years (*SD* = 10.62) with a mean of 41.15 working hours per week (*SD* = 8.48). More than 20 industry sectors were represented with the highest proportions stemming from health care and social assistance (13%), retail/trade (13%), manufacturing (10%), and construction (9%).

#### Measures

At time 1, we measured *affective job insecurity* using the 9-item scale developed by Probst (2003); response options ranged from 0 to 3, with higher scores indicating greater insecurity. Respondents were also asked to indicate if they had been *unemployed* at any point within the prior five years (dummy coded: 0 = No, 1 = Yes). At time 2, we measured *task-related self-efficacy* using an 8-item scale (Barbaranelli et al., 2018) with response options ranging from 1 (cannot do at all) to 7 (very certain I can do) for a self-evaluation outcome. Performance outcomes were assessed with the following three scales: *in-role task performance* (Williams & Anderson, 1991, 4-item scale ranging from 1—never to 5—always), *safety compliance* (Neal et al., 2000, 4-item scale ranging from 1—strongly disagree to 7—strongly agree), and *cognitive failures* (Wallace & Chen, 2005, 15-item scale ranging from 1—never to 5—very often). Items can be seen in our OSF folder.

### Study 2 Results

Means, standard deviations, correlations, and reliability coefficients are presented in Table S2. Corroborating earlier research, affective job insecurity was negatively related to task-related self-efficacy (*r* = − 0.26, *p* < 0.001) as a self-evaluation outcome, negatively related to in-role task performance (*r* = − 0.22, *p* < 0.001) and safety compliance (*r* = − 0.19, *p* < 0.001), and positively related to cognitive failures (*r* = 0.25, *p* < 0.001) as performance-related outcomes. Before testing our hypotheses, CFAs were conducted to ensure that the proposed measurement model fit the data adequately and compared it with alternative measurement models. These model comparison results (see Table S3) verified that measures used in the current study captured distinct constructs. In other words, the proposed five-factor model, whereby job insecurity (T1), task-related self-efficacy (T2), in-role task performance (T2), safety compliance (T2), and cognitive failures (T2), all represented distinct constructs, best represented the data [χ^2^(730) = 1758.67, *p* < 0.001, CFI = 0.91; RMSEA = 0.06; SRMR = 0.05]. More detailed model comparison results can be found in Table S3.

**Fig. 3:**
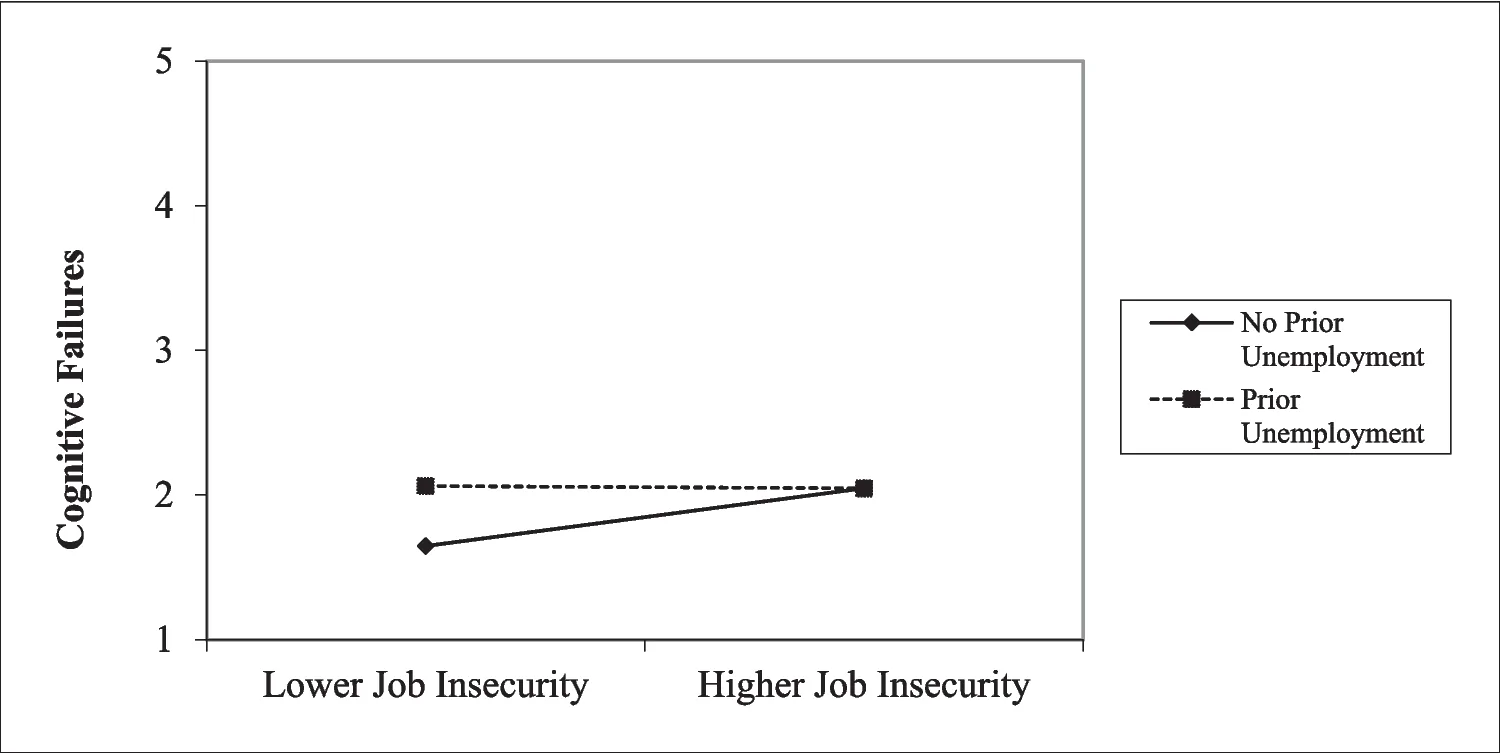
Prior unemployment moderates the job insecurity–cognitive failures relationship (study 2). Lower and higher refer to + 1 and − 1 SD

To test our hypothesized relationships, job insecurity was mean centered (Cohen et al., 2003), and the interaction term was created with the product of centered job insecurity and prior unemployment. Parameter estimates, standard errors, and *p*-values are reported in Table 3. Regarding the interaction hypotheses, our results showed significant job insecurity × prior unemployment interaction terms for the three performance-related outcomes: cognitive failures (interaction *γ* = − 0.21, *p* = 0.004), in-role task performance (interaction *γ* = 0.17, *p* = 0.042), and safety compliance (interaction *γ* = 0.25, *p* = 0.032), and for task-related self-efficacy (interaction *γ* = 0.21, *p* = 0.048) as a self-evaluation outcome. Simple slope tests revealed that the relationships of job insecurity with cognitive failures, in-role task performance, safety compliance, and task-related self-efficacy were significant for those who had no unemployment spells in the past 5 years (*γ* = 0.20, *p* < 0.001; *γ* = − 0.21, *p* < 0.001; *γ* = − 0.25, *p* < 0.001, and *γ* = −0.30, *p* < 0.001, respectively), but that these relationships were non-significant for those who had prior unemployment experiences (*γ* = − 0.01, *p* = 0.892; *γ* = − 0.04, *p* = 0.539; *γ* = − 0.01, *p* = −0.932, and *γ* = − 0.09, *p* = 0.337, respectively). The interaction effects are depicted in Figs. 3, S3, S4, and S5, respectively. As prior unemployment experiences buffered the impact of affective job insecurity on the three performance outcomes and on task-related self-efficacy, these findings support the stress inoculation perspective (Hypothesis 1b).

**Table 3:** Results from study 2

Outcome	Outcome	Parameter estimate	*SE*	*p*
*Self-evaluation outcome*	*Task-related self-efficacy*			
	Intercept	5.92	0.05	< 0.001
	Prior UE	− 0.13	0.12	0.273
	Job insecurity	− 0.30	0.06	< 0.001
	UE × job insecurity	0.21	0.11	0.048
	* R*^2^	0.08		
*Performance outcomes*	*In-role task performance*			
	Intercept	4.44	0.04	< 0.001
	Prior UE	− 0.01	0.09	0.956
	Job insecurity	− 0.21	0.04	< 0.001
	UE × job insecurity	0.17	0.08	0.042
	* R*^2^	0.06		
	*Safety compliance*			
	Intercept	6.06	0.06	< 0.001
	Prior UE	− 0.06	0.13	0.643
	Job insecurity	− 0.25	0.06	< 0.001
	UE × job insecurity	0.25	0.12	0.032
	* R*^2^	0.05		
	*Cognitive failures*			
	Intercept	1.85	0.04	< 0.001
	Prior UE	0.21	0.08	0.009
	Job insecurity	0.20	0.04	< .0001
	UE × job insecurity	− 0.21	0.07	0.004
	* R*^2^	0.09		

*N* = 390. Parameter estimates are unstandardized

*SE* standard error, *UE* unemployment experiences (0 = no and 1 = yes in past 5 years)

## Study 3: 2-Wave Dataset in USA (*N* = 575)

Building on study 2 results, we sought to replicate these in a larger US sample and also considered a broader set of outcomes. Of note, we used a continuous operationalization of unemployment experiences, allowing for a more nuanced measurement of prior spells.

### Study 3 Method

#### Participants

The second US dataset (*N* = 575) was collected via Amazon MTurk at two time points with a 1-month lag in 2018 and using the same pre-screening qualifications described above for quality control. Participants were compensated $3 for completion of each survey. The sample was predominantly male (65%). The average age of respondents was 34.42 years (*SD* = 9.77) with a mean working hours of 39.76 (*SD* = 9.26). More than 20 industry sectors were represented, with the highest proportions stemming from finance and insurance (10%), professional, scientific, and technical services (10%), retail (9%), and manufacturing (9%).

#### Measures

At time 1, we measured *affective job insecurity* again using the scale by Probst (2003). *Prior unemployment history* was quantified as the total number of unemployment spells over the past 5 years (*M* = 0.75, *SD* = 1.17). At time 2, we again measured *cognitive failures*, *safety compliance*, and *in-role task performance* using the same scales described for study 2. Additionally, we assessed both *positive and negative job-related affective well-being* (Van Katwyk et al., 2000, 12 items ranging from 1—never to 5—extremely often), *sleep disturbances* (Kecklund & Åkerstedt, 1992, 6 items ranging from 1—never to 5—very often), and *physical health conditions* (Hanisch, 1992, 13 items with each health problem dummy coded 0 = no and 1 = yes). Again, items can be seen in our OSF folder.

### Study 3 Results

Means, standard deviations, correlations, and reliability coefficients are presented in Table S4. Corroborating earlier research, affective job insecurity was negatively related to positive job-related affective well-being (*r* = − 0.35, *p* < 0.001) as an affective outcome, and negatively related to in-role task performance (*r* = − 0.15, *p* < 0.001) and safety compliance (*r* = − 0.26, *p* < 0.001) as performance-related outcomes. Moreover, job insecurity was positively associated with negative job-related affective well-being (*r* = 0.50, *p* < 0.001) as an affective outcome, cognitive failures (*r* = 0.37, *p* < 0.001) as a performance-related outcome, and physical health conditions (*r* = 0.27, *p* < 0.001) and sleep disturbances (*r* = 0.39, *p* < 0.001) as health-related outcomes. Before testing our hypotheses, CFAs were conducted to ensure that the proposed measurement model fit the data adequately and compared it with alternative measurement models. These model comparison results (see Table S3) verified that measures used in the current study captured distinct constructs. Put differently, the proposed eight-factor model, in which job insecurity (T1), negative job-related affective well-being (T2), positive job-related affective well-being (T2), in-role task performance (T2), safety compliance (T2), cognitive failures (T2), health conditions (T2), and sleep disturbances (T2) all represented distinct constructs, provided the best fit for the data [*χ*^2^(377) = 975.96, *p* < 0.001, CFI = 0.95; RMSEA = 0.05; SRMR = 0.06]. More detailed model comparison results can be found in Table S3.

**Fig. 4:**
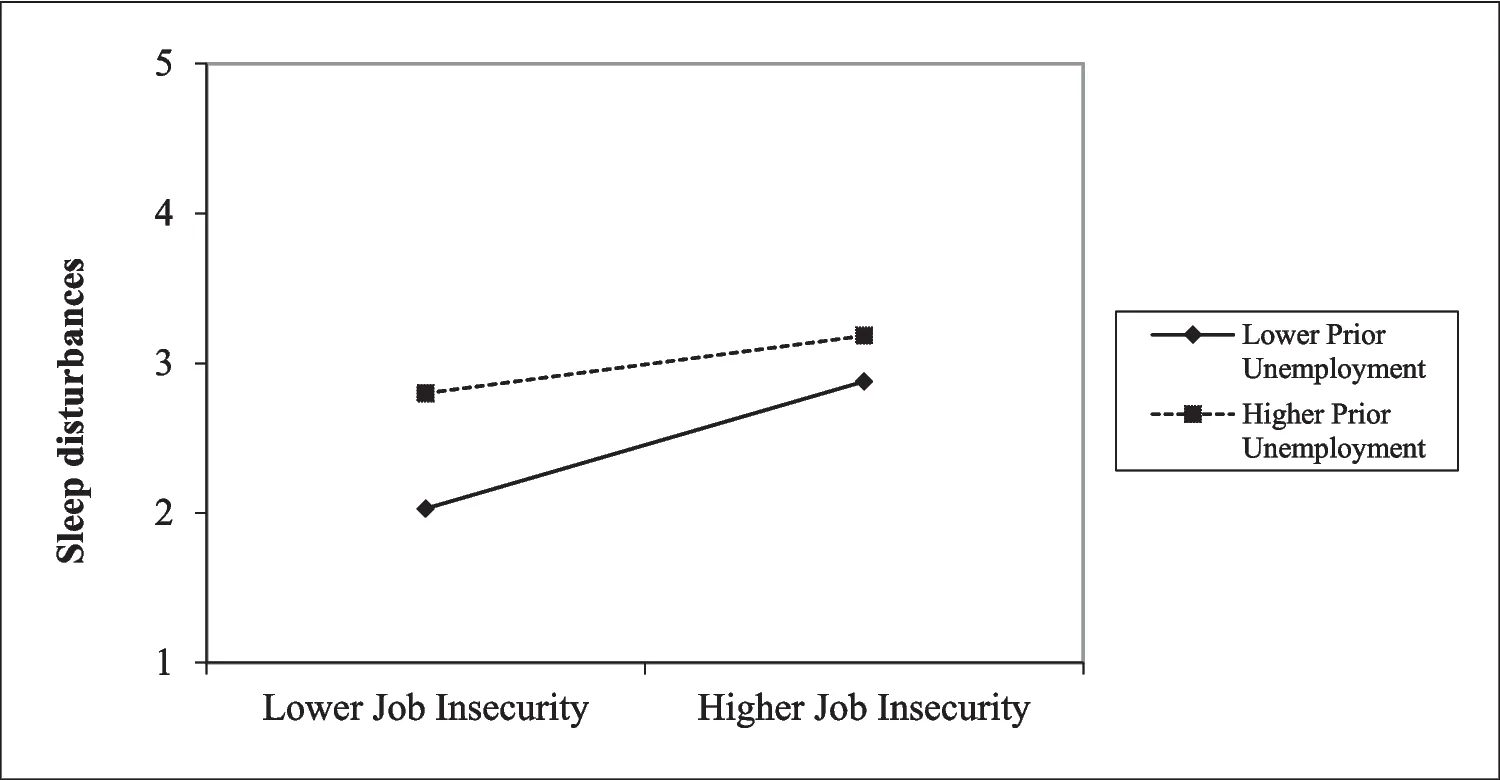
Prior unemployment moderates the job insecurity–sleep disturbances relationship (study 3). Lower and higher refer to + 1 and − 1 SD

To test our hypothesized relationships, job insecurity and prior unemployment history were mean centered (Cohen et al., 2003), and the interaction term was created with the product of centered job insecurity and prior unemployment. Parameter estimates, standard errors, and *p*-values are reported in Table 4. Regarding the interaction hypotheses, our results showed significant job insecurity × prior unemployment interaction terms for negative job-related affective well-being (interaction *γ* = −0.12, *p* = 0.004) as an affective outcome, in-role task performance (interaction *γ* = 0.07, *p* = 0.019) as a performance-related outcome, and sleep disturbances (interaction *γ* = − 0.10, *p* = 0.017) as a health outcome. More precisely, simple slope tests showed that among individuals who experienced more spells of unemployment, job insecurity was less strongly related to negative job-related affective well-being (*γ* = 0.30, *p* < 0.001), unrelated to in-role task performance (*γ* = 0.002, *p* = 0.966), and less strongly related to sleep disturbances (*γ* = 0.20, *p* = 0.004), while for those with fewer unemployment spells, job insecurity was more strongly related to negative job-related affective well-being (*γ* = 0.57, *p* < 0.001), significantly negatively related to in-role task performance (*γ* = − 0.16, *p* < 0.001), and more strongly related to sleep disturbances (*γ* = 0.44, *p* < 0.001). These moderation patterns are graphically depicted in Figures S6, S7, and Fig. 4. As earlier spells of unemployment buffered the relationships between job insecurity and the aforementioned outcomes, these findings lend support for the stress inoculation perspective (Hypothesis 1b). No significant interactions were found for positive job-related affective well-being, safety compliance, cognitive failures, or health conditions.

**Table 4:** Results from study 3

Outcome	Outcome	Parameter estimate	*SE*	*p*
*Affective outcomes*	*JAWS—positive*			
	Intercept	3.19	0.04	< 0.001
	Prior UE	0.17	0.03	< 0.001
	Job insecurity	− 0.39	0.04	< 0.001
	UE × job insecurity	0.03	0.04	0.447
	*R*^2^	0.17		
	*JAWS—negative*			
	Intercept	2.35	0.04	< 0.001
	Prior UE	0.24	0.03	< 0.001
	Job insecurity	0.43	0.04	< 0.001
	UE × job insecurity	− 0.12	0.04	0.004
	*R*^2^	0.31		
*Performance outcomes*	*In-role task performance*			
	Intercept	4.35	0.03	< 0.001
	Prior UE	− 0.05	0.02	0.044
	Job insecurity	− 0.08	0.03	0.004
	UE × job insecurity	0.07	0.03	0.019
	*R*^2^	0.04		
	*Safety compliance*			
	Intercept	5.94	0.06	< 0.001
	Prior UE	− 0.02	0.05	0.681
	Job insecurity	− 0.24	0.06	< 0.001
	UE × job insecurity	0.09	0.06	0.160
	*R*^2^	0.08		
	*Cognitive failures*			
	Intercept	2.23	0.06	< 0.001
	Prior UE	0.36	0.04	< 0.001
	Job insecurity	0.24	0.06	< 0.001
	UE × job insecurity	− 0.09	0.06	0.097
	*R*^2^	0.25		
*Health outcomes*	*Health conditions*			
	Intercept	2.86	0.14	< 0.001
	Prior UE	1.19	0.12	< 0.001
	Job insecurity	0.51	0.13	< 0.001
	UE × job insecurity	− 0.24	0.14	0.087
	*R*^2^	0.22		
	*Sleep disturbances*			
	Intercept	2.72	0.04	< 0.001
	Prior UE	0.23	0.04	< 0.001
	Job insecurity	0.32	0.04	< 0.001
	UE × job insecurity	− 0.10	0.04	0.017
	*R*^2^	0.21		

*N* = 193–575. Parameter estimates are unstandardized

*SE* standard error, *JAWS* job-related affective well-being scale, *UE* the number of prior unemployment spells in the previous 5 years

## Study 4: 3-Wave Dataset in Germany (*N* = 453)

In the last study, we further extended our analysis by investigating two posited mediating mechanisms (frustration and relative deprivation) using a 3-wave dataset.

### Study 4 Method

#### Participants

Data were collected in Spring 2021 on three occasions with a one-month lag between each wave through Bilendi, a market research company (for a similar approach see Batinic et al., 2010; Debus et al., 2020). The inclusion criteria were the same as in study 1. At T2 and T3 we also assessed whether participants still held the same job as an inclusion criterion. In total, 812 participants completed the time 1 survey, that is, they provided completed surveys, did not fail attention checks, and provided ratings within a reasonable period (i.e., we excluded surveys that were completed within a too short time interval, thus possibly indicating careless responding, Bilendi, n.d.). During the course of the study, 359 participants dropped out due to nonresponse or because they did not pass quality checks at T2 or T3 and were thus excluded from the final sample. The final dataset consisted of 453 participants with complete data, leading to a response rate of 55.79%. In total, 48% of participants identified as female; the average age was 45.04 years (*SD* = 11.95), and 88.1% of respondents indicated they worked in a full-time position. Participants were from 13 different industry types, including agriculture (1.3%), manufacturing (13.9%), health and social services (13.2%), and public services (12.1%).

#### Measures

If not indicated otherwise, all measures used a rating scale ranging from 1 = *strongly disagree* to 7 = *strongly agree*. At T1, we assessed *cognitive job insecurity* (Borg, 1992, 4-item scale) and *prior unemployment* (coded 0 = no prior unemployment, 1 = prior unemployment). At T2, we assessed *frustration* (Peters et al., 1980, 3-item scale) and *relative deprivation* (Callan et al., 2011, 3-item scale, adapted to the work situation, see Oki, 2013). At T3, we assessed the self-evaluative outcomes of *perceived demands-abilities fit* and *needs-supplies fit* (Luksyte et al., 2011, four items each, based on Cable & Judge, 1996; and Saks & Ashforth, 1997, respectively), the attitudinal outcomes *job satisfaction* (3-item job satisfaction subscale from the Michigan Organizational Assessment Questionnaire, MOAQ-JSS, Cammann et al., 1983), *intentions to remain* to operationalize turnover intention (Armstrong-Stassen & Ursel, 2009, 3-item scale), and the performance outcomes *task performance* (Staufenbiel & Hartz, 2000, 5-item scale, a German-language version of the scale by Williams & Anderson, 1991), and *incivility towards coworkers* (Porath & Pearson, 2012, 4-item scale). The latter measure originally assessed perceived incivility by the victim; however, for this study, we adapted it to measure instigated incivility.

### Study 4 Results

Means, standard deviations, and correlations of the study variables are displayed in Table S5. Corroborating earlier research (see Table S5), job insecurity was negatively related to demands-abilities fit (*r* = − 0.32, *p* < 0.001) and needs–supplies fit (*r* = − 0.31, *p* < 0.001) as self-evaluation outcomes, negatively related to intentions to remain (*r* = − 0.40, *p* < 0.001) and job satisfaction (*r* = − 0.37, *p* < 0.001) as attitudinal outcomes, negatively related to task performance (*r* = − 0.34, *p* < 0.001), and positively related to incivility (*r* = 0.18, *p* < 0.001)—both representing performance-related outcomes. Moreover, job insecurity was positively related to frustration (*r* = 0.33, *p* < 0.001) and relative deprivation (*r* = 0.39, *p* < 0.001). Before testing our hypotheses, CFAs were conducted to ensure that the proposed measurement model fit the data adequately and compared it with alternative measurement models. These model comparisons verified that measures used in the current study captured distinct constructs. In other words, the proposed nine-factor model, whereby job insecurity (T1), frustration (T2), relative deprivation (T2), perceived demands–abilities fit (T3), perceived needs–supplies fit (T3), intentions to remain (T3), job satisfaction (T3), task performance (T3), and incivility towards coworkers (T3) all represented distinct constructs, best represented the data [*χ*^2^(459) = 1283.297, *p* < 0.001, CFI = 0.94; RMSEA = 0.06; SRMR = 0.06]. More detailed model comparison results can be found in Table S3.

We applied multivariate path modeling to estimate the hypothesized pathways to test our hypotheses. Specifically, job insecurity was mean-centered (Cohen et al., 2003), and the product of centered job insecurity and prior unemployment (0 = no, 1 = yes) was used as the interaction term. Frustration and relative deprivation (i.e., mediators) were regressed on job insecurity, prior unemployment, and the interaction term. Perceived demands-abilities fit, perceived needs-supplies fit, job satisfaction, intentions to remain, task performance, and incivility toward coworkers (i.e., outcomes) were regressed on frustration, relative deprivation, job insecurity, and prior unemployment. We additionally controlled for the effects of the interaction term on all outcome variables (see Liu et al., 2012).

**Fig. 5:**
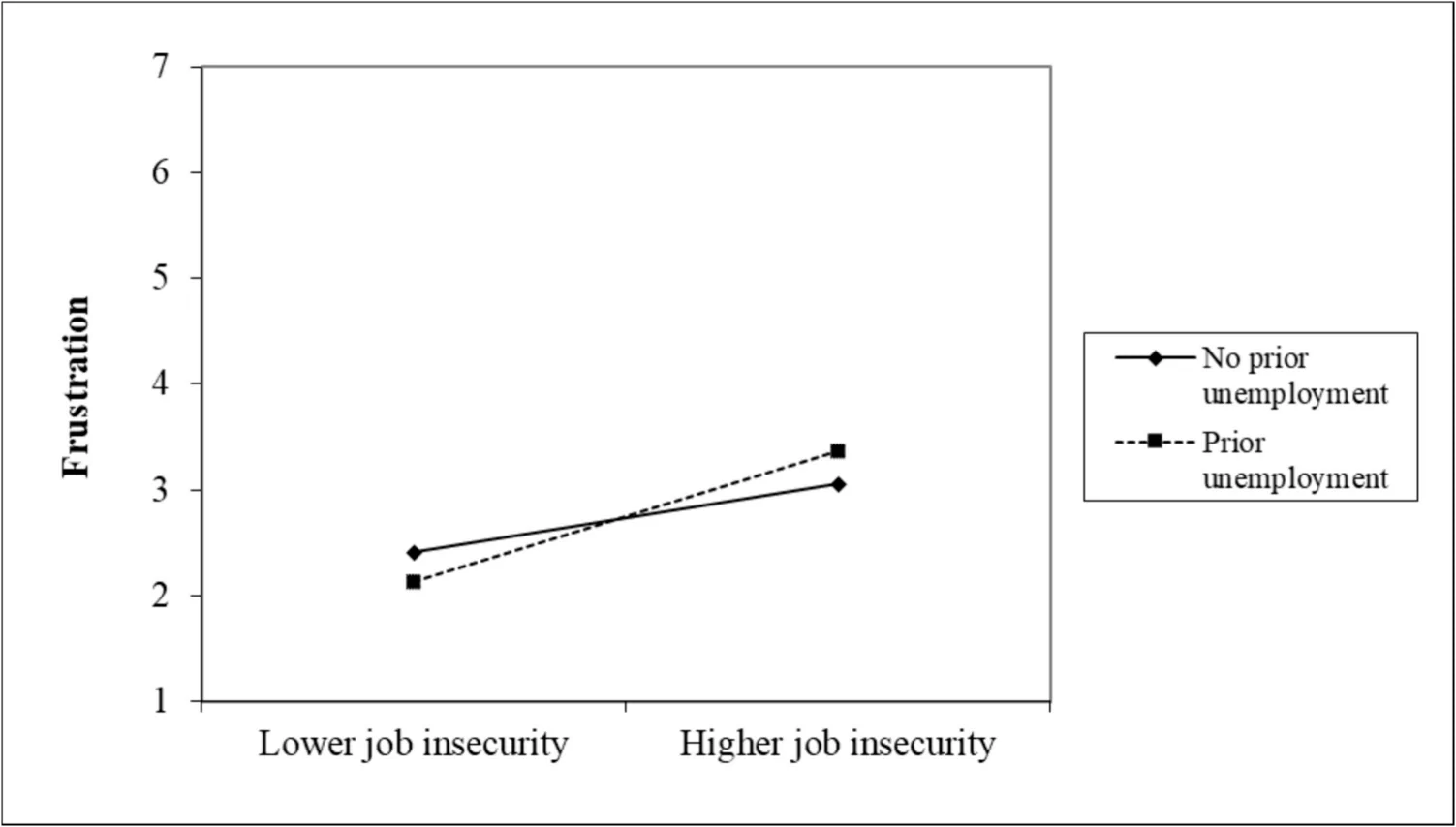
Prior unemployment moderates the job insecurity–frustration relationship (study 4)**.** Lower and higher refer to + 1 and − 1 SD

**Fig. 6:**
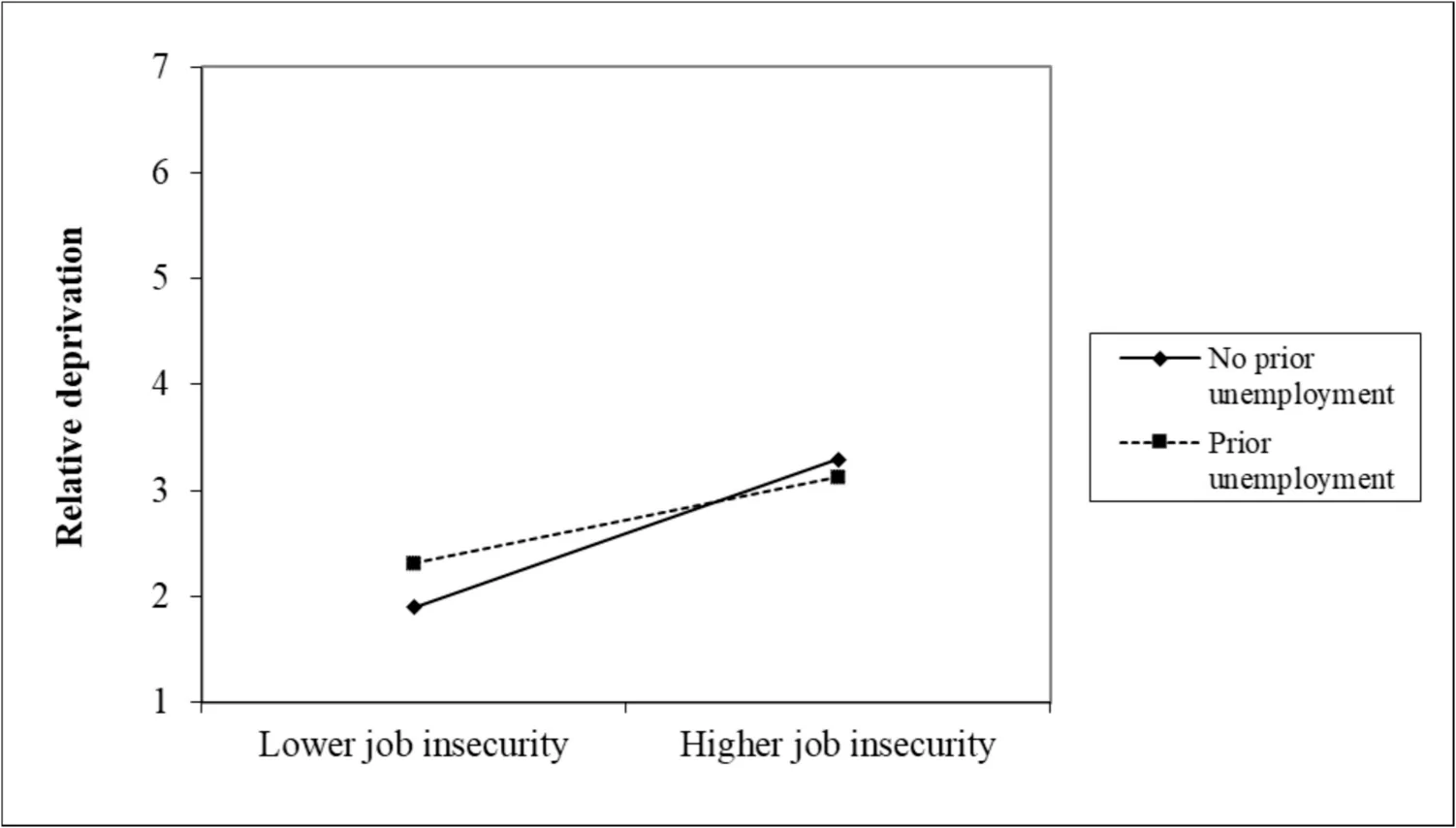
Prior unemployment moderates the job insecurity–relative deprivation relationship (study 4)*.* Lower and higher refer to + 1 and − 1 SD

Unstandardized parameter estimates are displayed in Table 5. As can be seen, the relationship between job insecurity and frustration was contingent on whether participants previously experienced unemployment (interaction *γ* = 0.24, *p* = 0.016). Figure 5 graphically displays the interaction effect. Simple slope tests showed that when individuals had experienced prior unemployment, job insecurity was positively related to frustration (*γ* = 0.50, *p* < 0.001). When employees had never experienced unemployment, job insecurity was more weakly related to frustration (*γ* = 0.26, *p* < 0.001). Second, the relationship between job insecurity and relative deprivation was contingent on whether participants previously experienced unemployment (interaction *γ* = − 0.24, *p* = 0.027, see Table 5). Figure 6 graphically displays the interaction effect. Simple slope tests showed that when individuals had experienced prior unemployment, job insecurity was positively related to relative deprivation (*γ* = 0.33, *p* < 0.001). Yet, among employees who had never experienced unemployment, job insecurity was even more strongly related to relative deprivation (*γ* = 0.56, *p* < 0.001). These findings provide preliminary support for our proposition that prior unemployment strengthens the relationship between job insecurity and frustration while also attenuating the relationship between job insecurity and relative deprivation.

**Table 5:** Unstandardized coefficients of the moderated mediation path model (study 4)

	Frustration			Relative deprivation			Perceived demands-abilities fit			Perceived needs-supplies fit			Intention to remain			Job satisfaction			Task performance			Incivility		
Variables	Est	*p*	*SE*	Est	*p*	*SE*	Est	*p*	*SE*	Est	*p*	*SE*	Est	*p*	*SE*	Est	*p*	*SE*	Est	*p*	*SE*	Est	*p*	*SE*
*Intercept*	2.73		0.08	2.59		0.09	6.88		0.10	7.06		0.13	7.11		0.16	7.47		0.12	6.62		0.09	1.03		0.10
*Independent variable*																								
Job insecurity	0.26	< 0.001	0.06	0.56	< 0.001	0.07	− 0.07	.079	0.04	− 0.06	.298	0.05	− 0.22	0.001	0.06	− 0.13	0.013	0.05	− 0.13	0.001	0.04	0.06	.139	0.04
Prior unemployment	0.02	.894	0.12	0.12	0.347	0.13	0.05	0.531	0.07	− 0.16	.102	0.10	− 0.13	0.254	0.12	− 0.16	0.074	0.09	0.11	0.109	0.07	− 0.06	.440	0.08
*Interaction*																								
Job insecurity × prior unemployment	0.24	0.016	0.10	− 0.24	0.027	0.11	− 0.07	0.266	0.06	− 0.08	.311	0.08	− 0.10	0.304	0.10	− 0.00	0.982	0.08	− 0.05	0.401	0.06	− 0.03	.613	0.06
*Mediators*																								
Frustration							− 0.17	< 0.001	0.03	− 0.36	< 0.001	0.04	− 0.35	< 0.001	0.05	− 0.50	< 0.001	0.04	− 0.13	< 0.001	0.03	0.15	< 0.001	0.03
Relative deprivation							− 0.14	< 0.001	0.03	− 0.20	< 0.001	0.04	− 0.20	< 0.001	0.05	− 0.19	< 0.001	0.04	−0.05	.051	0.03	0.05	.114	0.03
*R*^2^	11.9%			16.3%			26.8%			35%			33.4%			48.7%			19.2%			10.3%		

*N* = 453. Est. = parameter estimate, *SE *standard error

^1^For prior unemployment: no prior unemployment = 0; prior unemployment = 1

To test Hypotheses 2 and 3, we estimated conditional indirect effects at low vs. high values of the moderator (i.e., no prior unemployment vs. prior unemployment) and conducted formal tests of corresponding differences between these conditional indirect effects following common recommendations (Hayes, 2022; Preacher et al., 2007). We display point estimates and the respective 95% confidence intervals in Table 6. First, in line with our hypotheses, results showed a significant difference between values of conditional indirect effects for no prior unemployment vs. prior unemployment for the relationship between job insecurity and all six outcomes through *frustration* [difference between conditional indirect effects for perceived demands-abilities fit *γ* = − 0.04 [− 0.08, − 0.01]; for perceived needs-supplies fit *γ* = − 0.08 [− 0.16, − 0.02]; for intentions to remain *γ* = − 0.08 [− 0.16, − 0.02]; for job satisfaction *γ* = − 0.12 [− 0.22, − 0.02]; for task performance *γ* = − 0.03 [− 0.06, − 0.01]; for incivility towards coworkers *γ* = 0.04 [0.01, 0.07]. In sum, job insecurity was negatively and significantly related to perceived demands-abilities fit, perceived needs-supplies fit, intentions to remain, job satisfaction, and task performance via frustration for individuals without and with prior unemployment. Yet, the conditional indirect effects for individuals with prior unemployment were significantly stronger than those without prior unemployment. For incivility towards coworkers, both conditional indirect effects were significant and positive, yet the conditional indirect effect for individuals with prior unemployment was significantly stronger as compared to the conditional indirect effect for individuals without prior unemployment. Taken together, results indicate that job insecurity was more (vs. less) strongly related to all outcomes via frustration among employees with (vs. without) prior unemployment. Thus, Hypothesis 2 was supported.

**Table 6:** Unstandardized estimates and bias-corrected confidence intervals of indirect path coefficients of the moderated mediation model (study 4)

		Indirect effect
Variables	Estimate	95% CI [LL;UL]
**Job insecurity →** ***frustration* → perceived demands-abilities fit**		
* Indirect effect*	** − .05**	**[− .07, − .02]**
* Moderator prior unemployment: conditional indirect effects*		
Prior unemployment (PU)	** − .09**	**[− .13, − .05]**
No prior unemployment (NPU)	** − .05**	**[− .07, − .02]**
Difference between PU and NPU conditions	** − .04**	**[− .08, − .01]**
**Job insecurity → *frustration* → perceived needs-supplies fit**		
* Indirect effect*	** − .09**	**[− .14, − .05]**
* Moderator prior unemployment: conditional indirect effects*		
Prior unemployment (PU)	** − .18**	**[− .25, − .11]**
No prior unemployment (NPU)	** − .09**	**[− .14, − .05]**
Difference between PU and NPU conditions	** − .08**	**[− .16, − .02]**
**Job insecurity → *frustration* → intentions to remain**		
* Indirect effect*	** − .09**	**[− .14, − .04]**
* Moderator prior unemployment: conditional indirect effects*		
Prior unemployment (PU)	** − .17**	**[− .25, − .11]**
No prior unemployment (NPU)	** − .09**	**[− .15, − .05]**
Difference between PU and NPU conditions	** − .08**	**[− .16, − .01]**
**Job insecurity → *frustration* → job satisfaction**		
* Indirect effect*	** − .13**	**[− .20, − .07]**
* Moderator prior unemployment: conditional indirect effects*		
Prior unemployment (PU)	** − .25**	**[− .34, − .17]**
No prior unemployment (NPU)	** − .13**	**[− .20, − .07]**
Difference between PU and NPU conditions	** − .12**	**[− .22, − .02]**
**Job insecurity → *frustration* → task performance**		
* Indirect effect*	** − .03**	**[− .06, − .01]**
* Moderator prior unemployment: conditional indirect effects*		
Prior unemployment (PU)	** − .07**	**[− .10, − .03]**
No prior unemployment (NPU)	** − .03**	**[− .06, − .01]**
Difference between PU and NPU conditions	** − .03**	**[− .06, − .01]**
**Job insecurity → *frustration*** **→ incivility towards coworkers**		
* Indirect effect*	**.04**	**[.02, .07]**
* Moderator prior unemployment: conditional indirect effects*		
Prior unemployment (PU)	**.07**	**[.04, .12]**
No prior unemployment (NPU)	**.04**	**[.02, .07]**
Difference between PU and NPU conditions	**.04**	**[.01, .07]**
**Job insecurity → *relative deprivation* → perceived demands-abilities fit**		
* Indirect effect*	**-.08**	**[-.12, -.05]**
* Moderator prior unemployment: conditional indirect effects*		
Prior unemployment (PU)	**-.05**	**[-.08, -.02]**
No prior unemployment (NPU)	**-.08**	**[-.12, -.05]**
Difference between PU and NPU conditions	**.03**	**[.003, .07]**
**Job insecurity → *relative deprivation* → perceived needs-supplies fit**		
* Indirect effect*	**-.11**	**[-.15, -.07]**
* Moderator prior unemployment: conditional indirect effects*		
Prior unemployment (PU)	**-.06**	**[-.11, -.03]**
No prior unemployment (NPU)	**-.11**	**[-.16, -.06]**
Difference between PU and NPU conditions	**.05**	**[.005, .09]**
**Job insecurity →** ***relative deprivation*** **→ intentions to remain**		
* Indirect effect*	**-.11**	**[-.16, -.07]**
* Moderator prior unemployment: conditional indirect effects*		
Prior unemployment (PU)	**-.07**	**[-.11, -.03]**
No prior unemployment (NPU)	**-.11**	**[-.17, -.06]**
Difference between PU and NPU conditions	**.05**	**[.005, .10]**
**Job insecurity → *relative deprivation* → job satisfaction**		
* Indirect effect*	**-.11**	**[-.15, -.07]**
* Moderator prior unemployment: conditional indirect effects*		
Prior unemployment (PU)	**-.06**	**[-.10, -.03]**
No prior unemployment (NPU)	**-.11**	**[-.16, -.06]**
Difference between PU and NPU conditions	**.04**	**[.005, .09]**
**Job insecurity → *relative deprivation* → task performance**		
* Indirect effect*	-.03	[-.06, .002]
* Moderator Prior unemployment: Conditional indirect effects*		
Prior unemployment (PU)	-.02	[-.04, .0005]
No prior unemployment (NPU)	-.03	[-.06, .0005]
Difference between PU and NPU conditions	.01	[-.001, .03]
**Job insecurity → *relative deprivation* → incivility towards coworkers**		
* Indirect effect*	.03	[-.004, .06]
* Moderator prior unemployment: conditional indirect effects*		
Prior unemployment (PU)	.02	[-.003, .04]
No prior unemployment (NPU)	.03	[-.007, .06]
Difference between PU and NPU conditions	-.01	[-.03, .003]

*N* = 453. Parameter estimates are unstandardized. 95% CI refers to the Monte Carlo 95% confidence interval with 20,000 draws (Selig & Preacher, 2008)

*LL* lower limit, *UL* upper limit

Bolded estimates represent significant effects

Second, results showed a significant difference between values of conditional indirect effects for no prior unemployment vs. prior unemployment for the relationship between job insecurity and four (perceived demands-abilities fit, perceived needs-supplies fit, intentions to remain, job satisfaction) out of six outcomes through *relative deprivation* (difference between conditional indirect effects for perceived demands-abilities fit *γ* = 0.03 [0.004, 0.07], perceived needs-supplies fit *γ* = 0.05 [0.005, 0.09], intentions to remain *γ* = 0.05 [0.005, 0.10], and job satisfaction *γ* = 0.04 [0.01, 0.09]). More precisely, job insecurity was negatively and significantly related to perceived demands-abilities fit, perceived needs-supplies fit, intentions to remain, and job satisfaction via relative deprivation for individuals without and with prior unemployment. For all these outcomes, the conditional indirect effects for individuals with prior unemployment were significantly weaker as compared to individuals without prior unemployment. For task performance and incivility towards coworkers, both conditional indirect effects as well as the difference between conditional indirect effects were nonsignificant (difference between conditional indirect effects for task performance *γ* = 0.01 [− 0.001, 0.03], for incivility towards coworkers *γ* = − 0.01 [− 0.03, 0.003]). The results indicate that job insecurity was less (vs. more) strongly related to four out of six outcomes via relative deprivation among employees with (vs. without) prior unemployment. Thus, Hypothesis 3 was supported for perceived demands-abilities fit, perceived needs-supplies fit, intentions to remain, and job satisfaction.

## Discussion

### Summary of Findings

The overarching goal of this study was to empirically compare two theoretical perspectives—stress sensitization and stress inoculation—derived from COR and TTSC, respectively, to determine how and the mechanisms by which prior unemployment affects reactions to current job insecurity perceptions. We tested our assumptions in four different studies, the results of which are summarized in Table 7.

**Table 7:** Summary of significant results

	Mechanisms			
	H1a: Stress sensitization	H1b: Stress inoculation	H2: Frustration	H3: Relative deprivation
*Self-evaluation outcomes*				
Perceived demands-abilities fit			✓(Study 4)	✓ (Study 4)
Perceived needs-supplies fit			✓ (Study 4)	✓ (Study 4)
Task-related self-efficacy		✓ (Study 2)		
*Affective outcomes*				
Negative affect				
Affective well-being		✓ (Study 3)		
*Attitudinal outcomes*				
Job satisfaction	✓ (Study 1)		✓ (Study 4)	✓ (Study 4)
Turnover intention	✓ (Study 1)			
Intentions to remain			✓ (Study 4)	✓ (Study 4)
Career commitment	✓ (Study 1)			
*Performance outcomes*				
Task performance		✓ (Studies 2 & 3)	✓ (Study 4)	
Incivility			✓ (Study 4)	
Safety compliance		✓ (Study 2)		
Cognitive failures		✓ (Study 2)		
*Health outcomes*				
Mental health				
Physical health				
Sleep disturbances		✓ (Study 3)		

Moderation hypotheses regarding the impact of prior unemployment experiences on self-evaluative, affective, attitudinal, performance, and health-related outcomes of job insecurity were tested in studies 1–3. First, utilizing data from employees in Germany, study 1 found evidence for a *stress sensitization* pattern for the attitudinal outcomes of job satisfaction, turnover intention, and career commitment such that the negative relationship between job insecurity and job satisfaction, the positive relationship between job insecurity and turnover intention, and the negative relationship between job insecurity and career commitment were stronger when individuals had experienced at least one unemployment spell. Second, utilizing two-wave data from employees in the US, study 2 supported the *stress inoculation* effect for performance outcomes (i.e., task performance, safety compliance, and cognitive failures) and for task-related self-efficacy as a self-evaluation outcome. Among individuals who had not experienced unemployment in the last 5 years, job insecurity was significantly negatively related to task performance and safety compliance, significantly positively related to cognitive failures, and significantly negatively related to task-related self-efficacy; yet, it was unrelated to all four outcomes among individuals who had experienced unemployment in the last 5 years. Third, utilizing two-wave data from employees in the US, study 3 also found evidence for a *stress inoculation* effect for job-related affective well-being (negative emotions) as an affective outcome, task performance as a performance outcome, and sleep disturbances as a health outcome. Among individuals with fewer unemployment spells, job insecurity was more strongly positively related to negative job-related affective well-being, significantly negatively related to in-role task performance, and more strongly positively related to sleep disturbances, whereas for those who experienced more unemployment spells, job insecurity was less strongly positively related to negative job-related affective well-being, unrelated to in-role task performance, and less strongly positively related to sleep disturbances.

Regarding the moderated mediation hypotheses, study 4, which utilized three-wave data from employees in Germany, found two parallel indirect effects of job insecurity on self-evaluative outcomes (i.e., perceived demands-abilities fit, perceived needs-supplies fit) and attitudinal outcomes (i.e., intentions to remain, job satisfaction) via *frustration* and *relative deprivation*. These effects were moderated by prior unemployment. More precisely, and in support of the stress sensitization perspective, prior unemployment strengthened the relationship between job insecurity and frustration. In support of the stress inoculation perspective, prior unemployment attenuated the relationship between job insecurity and relative deprivation. For the performance outcomes (i.e., task performance and incivility towards coworkers), we found evidence for a moderated mediation effect via frustration, but not via relative deprivation—thus supporting the stress-sensitization perspective.

A few findings are worthy of further scrutiny. The stress sensitization perspective was empirically supported by findings in studies 1 and 4 (via frustration), whereas the stress inoculation perspective received empirical support from findings of studies 2, 3, and 4 (only for self-evaluative and attitudinal outcomes via relative deprivation). Two considerations may help explain this pattern of findings: First, studies 1 and 4 were based on samples collected in Germany, whereas studies 2 and 3 analyzed data collected in the US. It is possible that contextual differences in social security and safety nets might account for these observed differences, given prior empirical evidence that state- and country-level variables affect individual-level processes (e.g., Debus et al., 2012; Probst et al., 2020a, 2020b). In particular, it might be the case that US employees are more likely to demonstrate the stress inoculation (vs. the stress sensitization) pattern due to the relatively weak labor protections in the US (vs. in Germany) which render the experience of unemployment potentially more frequent but also more normative. For example, US data from the National Longitudinal Survey of Youth which began in 1979 found that workers experienced on average 5.9 spells of unemployment between the ages of 18 and 56 (U.S. Bureau of Labor Statistics, 2024), whereas estimates of individual unemployment risk in Germany for that same historical timeframe were quite low (Wilke, 2005). Thus, compared to their German counterparts, US employees might be more likely to build coping resources and adopt a more flexible mindset that accommodates career-related ups and downs, including spells of unemployment and periods of insecurity. Second, studies 1 and 4 data were collected during the COVID-19 pandemic, whereas studies 2 and 3 data were collected prior to the pandemic. As such, it is possible that the concurrent health and economic crisis might have been perceived as an additional stressor by participants (Sinclair et al., 2021), resulting in more negative perceptions of economic stressors that made stressor sensitization more likely. Future research is warranted to examine these explanations in more depth.

Finally, one may wonder why only the stress sensitization process via frustration (but not the stress inoculation process via relative deprivation) was supported for task performance and incivility toward coworkers in study 4. Conceptual mismatch between deprivation and these outcomes might be a possible explanation: relative deprivation has been described as an inward and affectively laden mechanism (Luksyte et al., 2022) whereby individuals compare their current situation with what they “want, expect, and feel entitled to” (p. 322); yet, task performance and incivility towards coworkers are outwardly directed behaviors towards the organization and work colleagues. The stress sensitization mechanism may thus be the more salient mechanism for these two outcomes.

### Theoretical Implications

#### The Value of a Strong Inference Approach to Theory Testing

While COR and TTSC both make similar predictions regarding the main effects of job insecurity, we utilized a strong inference approach (e.g., Platt, 1964) to more rigorously test the boundary conditions of these hypothesized effects to better understand when and for whom these main effects hold and to better discern the value of the two theories (Meehl, 1990). By comparing theoretically derived predictions, we were able to assess the degree of support for stress sensitization (derived from COR) versus stress inoculation (derived from TTSC) effects and their associated underlying theories. By systematically deriving and testing predictions from these distinct theoretical frameworks, we sought to ensure that our findings are not only empirically robust but also theoretically meaningful.

In doing so, we also reduce the risk of confirmation bias (Oswald & Grosjean, 2004), as strong inference testing requires explicit consideration of alternative explanations and their implications. This allowed us to examine the interplay between job insecurity and prior unemployment experiences in order to better understand when and why past experiences may lead to enhanced vulnerability vs. resilience. Evidence from study 1 supports the sensitization hypothesis for attitudinal outcomes, but the bulk of the evidence from studies 2 and 3 favored the stress inoculation hypothesis for self-evaluative, affective, performance, and health outcomes, whereas the findings from study 4 comparing explanatory mediating mechanisms indicated that the form of the interaction might depend on what is being triggered by perceived job insecurity (i.e., feelings of frustration vs. relative deprivation).

Thus, while our results across the four samples were admittedly somewhat mixed, collectively they caution against an overly simplistic reliance on either COR or TTSC. This is particularly evident when examining reactions to currently experienced stressors in the context of prior stressor exposures to ascertain when and why this prior stressor exposure will lead to increased vulnerability rather than resilience. While COR and TTSC are among the most frequently used theories by job insecurity researchers (e.g., Bazzoli & Probst, 2022) to predict and explain the *commonly observed main effects of job insecurity*, our data revealed that a more nuanced perspective regarding these two theories is warranted. While negative self-evaluative, affective, attitudinal, performance, and health-related outcomes can be attributed to the threat of job loss (i.e., job insecurity), our interpretation of that threat *in the context of prior* experienced economic stress (i.e., prior spells of unemployment) also plays a crucial role. In some circumstances and in line with COR theory, prior loss results in a heightened stress response. Yet, our data showed that in other circumstances, consistent with TTSC, prior unemployment may affect how individuals perceive job insecurity (via their primary appraisal of the potential stressor including a more flexible mindset that considers periods of insecurity and unemployment as more normative) and evaluate their capacity to deal with the situation (via their secondary appraisal of their repertoire of coping skills).

#### The Importance of Incorporating Economic Stress Across the Life Course

Second, our study contributes a life course and temporal perspective to the study of job insecurity and economic stressors in general (Debus & Unger, 2024; Graham & Sinclair, 2024). Specifically, we consider how previous stressful career events, such as experiencing unemployment, can influence reactions to subsequent career challenges, such as job insecurity. In doing so, we enrich previous research that has, to date, mostly applied a contemporaneous perspective (see also Shipp & Jansen, 2011) where job insecurity–outcome relationships are shaped by individual, organizational, and societal factors happening at the same point in time (Jiang, 2025; Lee et al., 2018). Because stress experiences are inherently dynamic, employees’ previous experiences form a temporal context in which they are embedded. By explicitly considering this temporal context, we can achieve a more holistic understanding of the factors that shape individual stress reactions. In line with this, a pivotal study by Graham and Sinclair (2024) suggested that past adversities in life can propel later negative work attitudes and behaviors. While our study focused on the effects of unemployment and job insecurity as economic stressors, COR theory and TTSC suggest that these findings may be applicable to other types of economic stressors. Thus, there is potential for future research to explore and expand on this area of study.

In a related vein, our research also contributes to connecting the literature on economic stressors and the nascent literature on career shocks (e.g., Seibert et al., 2013), thus adding a career perspective to the study of economic stressors. Career shocks are “disruptive and extraordinary events that are, at least to some degree, caused by factors outside the focal individual’s control” (Akkermans et al., 2018, p. 4). These shocks are not isolated experiences, but they have implications for career trajectories, career plans, and later experiences (Seibert et al., 2013). Moreover, career shocks can have either positive or negative valence. For example, they can be a result of an unexpected promotion, but they can also be the outcome of negative events, such as unemployment or an ethical scandal within an organization. Indeed, the traditional career model of lifetime employment has become less likely due to changes in the labor market in recent decades (Akkermans et al., 2018, 2021). As a result, career disruptions and career shocks are becoming increasingly common, making it a pertinent phenomenon to study. Although there have been a few studies that have examined the direct impact of career shocks (i.e., negative organization-related event such as reduction in workforce; COVID-19 pandemic) on subsequent job insecurity (Hofer et al., 2021; Lin et al., 2021), our study contributes to this perspective by investigating how prior unemployment as a career shock can impact reactions to later job insecurity. We believe our findings can stimulate further research into this direction, providing valuable insights into the effects of career shocks on employees’ well-being and other work outcomes.

### Practical Implications

The strong inference approach used to evaluate how prior unemployment shapes reactions to job insecurity has practical implications and can inform the development of organizational interventions (e.g., by preventing stress sensitization through bolstering resources for at-risk employees and building resilience via training programs to enhance coping skills). Above all, our results indicate that organizations and managers should consider employees’ previous employment and career history in economically turbulent times. When estimating the threat that job insecurity may have on their employees, organizations may intuitively think of their employees’ *present and future* situation (e.g., their employability or ease of finding a new job, financial duties, and family obligations, Berntson et al., 2010; Elshaer et al., 2022). While these are important factors to consider, our study highlights that managers need to also consider their employee’s *past* unemployment histories in the context of personnel meetings, organizational communication strategies, and employee assistance programs (e.g., outplacement support).

Moreover, and as mentioned above, our study found significant interaction effects supporting both theories, but the applicability of each may depend on boundary conditions such as social benefits and economic environments. As such, we recommend that organizations (especially multinational companies) consider the social benefits, economic environments, and coping abilities when they need to go through structural changes.

Further, interventions related to social comparisons might also be helpful to avoid ruminating about perceived job insecurity, given that such rumination is usually associated with lower well-being (Holman & Silver, 1998). For example, research has shown that individuals under threat (i.e., cancer patients) prefer to interact with others who face the same situation, yet who are better off and to hear uplifting stories from these individuals (Molleman et al., 1986; Taylor & Lobel, 1989). It might be the case that individuals prefer these upward comparisons because more fortunate others can serve as role models and provide information about how to successfully cope with the illness. Applied to the present context, one may assume that if job insecure individuals interact with others who faced job insecurity or unemployment in the past, yet successfully mastered these situations, this can provide job insecure individuals with helpful information about how to successfully navigate and cope with career downturns and disruptions. Indeed, Danckert (2017) found that personal and vicarious (via close family members) experiences of unemployment positively altered one’s perceptions about unemployment. Outplacement agencies and career counselors may thus consider explicitly incorporating interactions with such role models when counseling job insecure individuals. Finally, practitioners should also consider that job insecure employees who previously experienced unemployment may feel more frustrated than those who have not experienced unemployment before. Therefore, again, we emphasize that these suggestions should not be taken as an invitation to disregard any explicit or implicit psychological contract responsibilities regarding expectations of job security, career development, and training to enhance future employability.

Finally, and corroborating earlier findings (see Cheng & Chan, 2008; Jiang & Lavaysse, 2018; Sverke et al., 2002, 2019, for meta-analyses), our research consistently identified significant relationships between job insecurity and the five key outcome domains, thus highlighting the detrimental impact of job insecurity. Clearly, job insecurity perceptions are due to characteristics of the objective situation as well as subjective characteristics of the individual (e.g., Keim et al., 2014), and not all of them can be easily changed. However, what organizations and managers can do is provide clear and honest communication to their employees about their future job situation, such that negative rumors (particularly when organizations are facing economically challenging times) are less likely to develop and spread, as well as avoid temporary contracts whenever possible (Debus et al., 2014; Jiang & Probst, 2014; Keim et al., 2014; Vander Elst et al., 2010).

### Limitations and Future Directions

Our research is not without limitations. First, while we acknowledge that we first developed the questions and hypotheses for this interesting line of research and subsequently identified prior archival datasets we had gathered within our research group to incrementally test our hypotheses, future research should adopt a more intentional approach. Further, while the use of archival datasets provided access to the major categories of job insecurity outcomes identified in prior meta-analyses (e.g., Jiang & Lavaysse, 2018), it did not always result in a “clean” narrative. However, cherry-picking outcomes to create a cleaner set of findings would have contributed to the file drawer problem and the perpetuation of the “Chrysalis effect” in organizational sciences (O’Boyle Jr et al., 2017). Nevertheless, we acknowledge that our selection and operationalization of variables was limited to what was available in our secondary data sources. While these secondary data sources revealed clear evidence for both sensitization and inoculation processes, future research is needed to determine why and under what conditions these different pathways may become salient. Our study 4 indicates that frustration and relative deprivation may be two explanatory mediating mechanisms for these effects; yet, unmeasured moderators and/or confounds (e.g., social safety net, historical timeframe differences, cultural values, outcome class) may explain our current mixed results and need to be more intentionally evaluated in future work on this topic.

Second, while our current study moved beyond a traditional contemporaneous focus to explore how prior experiences of unemployment predict currently experienced job insecurity, our approach was nonetheless *variable-centered*. As such, we can describe apparent relationships among the variables of interest (e.g., unemployment, job insecurity, and a host of known outcomes of job insecurity) with the aim of developing a parsimonious set of parameters generalizable to a larger population (Howard & Hoffman, 2018). Yet, it may be fruitful to consider *person-centered* approaches that acknowledge the fact that there may be sub-groups of individuals who differ in their configurations or patterns of certain characteristics and experiences (e.g., Woo et al., 2024). Latent profile analysis, for example, could test whether there are different profiles of individuals who may be more (or less) likely to experience frustration vs. relative deprivation–mediated effects. This might be based on personality characteristics (e.g., hope, optimism, resilience) or exposure to other forms of economic stress (e.g., unemployment spells, underemployment, financial precarity). Latent transition analysis, another form of person-centered analysis, might be used to create groups of individuals based on such profiles while considering their movement from one profile to another. Such an approach would allow for a much richer between- and within-person understanding of how the fabric of one’s prior employment history predicts reactions to currently experienced economic stressors. For example, Bazzoli et al. (2022) recently identified two latent profiles of precarity based on a variety of indicators of economic stressor exposure, including unemployment history, underemployment, job insecurity, household income, and financial strain. Membership in the precarious profile was adversely associated with numerous outcomes in work, life, and health domains. Extending this research could, for example, assess whether precarious profile workers are more likely to exhibit stress sensitization vs. stress inoculation effects, as well as whether profile membership is differentially related to frustration vs. relative deprivation as explanatory mechanisms.

Third, while we measured frustration and relative deprivation as two potential underlying mechanisms related to the stress sensitization and stress inoculation perspectives, a more nuanced assessment of the underlying theoretical processes is warranted. For example, future research could directly measure diminished resource pools (to better test the stress sensitization effect) and examine individuals’ self-referential comparisons, coping skills, and flexible mindsets regarding what constitutes a “normal” career trajectory (to better test the stress inoculation effect). Grounded in COR theory, we assumed that repeated actual losses of and threats of losing one’s employment status deplete resource pools (e.g., a shrinking network, reduced financial stability), leading to frustration. Conversely, based on TTSC, we assumed that individuals with prior unemployment experiences develop stronger coping skills and a more flexible mindset about employment trajectories, viewing disruptions and periods of insecurity and precarity as more normative and, therefore, are less likely to feel deprived in response to job insecurity (compare the seminal work by, e.g., Dweck & Yeager, 2019, on mindsets and their predictive validity for feelings and behavior). Hence, future research may more explicitly test these mechanisms to determine whether individuals’ diminished resource pools and their coping resources and mindsets serve as the key intermediate chain that explains variation in feelings of frustration and relative deprivation (respectively).

Fourth, future research may also consider macro-level conditions, such as cultural values, that could predispose individuals to displaying the stress sensitization vs. the stress inoculation mechanisms. Researchers have repeatedly highlighted the role of macro-level conditions in the context of economic stressors (e.g., Lee et al., 2018; Sinclair et al., 2024), making this a relevant aspect to study. As an example, the processes investigated in the current study might be affected by a country’s masculinity values as the extent to which traditionally more masculine attributes, such as being strong, assertive, having high status, and success are valued (Hofstede, 1998, 2001). In masculine countries, having a job is of central importance because it helps individuals to attain said masculine attributes (see also Debus et al., 2020). Applied to the context of our research, it is conceivable that individuals from countries high in masculinity are more likely to display the stress sensitization pattern, whereas individuals from countries low in masculinity may be more likely to display the stress inoculation pattern. Put differently, for individuals in highly masculine countries, being unemployed likely is a highly stressful situation that questions their preferred identity of being strong and successful—thus making them more likely to experience stress sensitization in the face of unemployment. In contrast, individuals in countries that score low on masculinity may be more likely to hold a mindset that incorporates disruptions and insecurity as part of a typical employment trajectory—because being constantly in the labor force is less central for their feelings of being successful. As such, individuals from countries with low levels of masculinity may be more likely to show an attenuated stress reaction to job insecurity in the context of previously experienced unemployment.

Fifth, we would encourage future researchers to use more nuanced conceptual and operational definitions of unemployment experiences. Much of the meta-analytic literature on unemployment (e.g., Amiri, 2022; Gedikli et al., 2023; McKee-Ryan et al., 2005; Nolte-Troha et al., 2023; Picchio & Ubaldi, 2024) either (a) failed to explicitly define unemployment, (b) did not differentiate between voluntary and involuntary unemployment episodes, and/or (c) used search terms (e.g., “job loss” but also “unemployed”) which could have returned studies of voluntary and involuntary episodes. Our study is not immune from this same limitation. While we explicitly were interested in unemployment regardless of the cause, our operationalization did not empirically assess whether the respondents’ episodes were voluntary and/or involuntary. Such information would have been useful to evaluate, for example, whether reactions to job insecurity are better/worse when prior experiences of unemployment were involuntary (rather than voluntary). Similarly, we only had information related to the experience (and in some studies, the number) of unemployment episodes. However, knowing the duration of these spells would also be important in specifying a comprehensive model of the potential interactions between unemployment and job insecurity. For example, longer unemployment durations might exacerbate the stress sensitization effects; yet, longer durations might also allow for more opportunity to develop and hone effective coping mechanisms in support of possible stress inoculation effects.

Finally, our study adopts a life course perspective by examining how a specific, identifiable past experience—unemployment—affects individuals’ later reactions to job insecurity. A strength of this approach is its focus on temporal sequencing, where earlier life events shape subsequent work experiences and perceptions. However, we acknowledge that certain boundary conditions that co-occur with job insecurity (e.g., coping strategies, Cheng et al., 2014; resilience, Shoss et al., 2018) may themselves be shaped by individuals’ accumulated past experiences. Although these factors are typically studied as concurrent moderators, they can also be understood as outcomes of a broader life course development. This raises an important distinction between studying the influence of specific past events and understanding how current psychological states have evolved over time. Future research could build on this by more systematically examining the developmental origins of such coping resources. Longitudinal designs that allow monitoring of individuals’ developmental pathways would be especially valuable for clarifying these dynamic pathways (Barling & Weatherhead, 2016; Kammeyer-Mueller et al., 2008). In a related vein, it is important to acknowledge that life course theory is even broader in scope (e.g., Elder & Shanahan, 2006; Thoits, 2010). The theory emphasizes not only the role of individual experiences over time, but also how these experiences are embedded within and shaped by larger social structures, historical contexts, and personal agency. By focusing primarily on the temporal ordering of employment experiences, our study addresses only a narrow slice of the life course framework. It does not fully capture, for example, how institutional policies, societal norms, or family networks may have influenced individuals’ trajectories or interpretations of job insecurity. Future research could more fully engage with life course theory by examining how linked lives (e.g., partners’ employment status), historical context (e.g., cohort differences in labor market entry), and agency (e.g., coping strategies and career planning) interact to shape the reactions to job insecurity and economic stress more broadly.

## Conclusion

In a set of four different studies, we examined the interplay between prior unemployment spells and current job insecurity on a plethora of key outcomes. In doing so, we applied a strong inference approach (Platt, 1964) by pitting stress sensitization versus stress inoculation as different theoretically derived predictions against each other and finding evidence that both inoculation (as seen in the study 2 and 3 US samples) and sensitization (as seen in the study 1 German sample) processes occur. Moreover, comparing explanatory mediating mechanisms in study 4 indicated that the form of the interaction might depend on what is being triggered by perceived job insecurity (i.e., feelings of frustration vs. relative deprivation). Given that unmeasured moderators and/or confounds (e.g., social safety net, historical timeframe differences, cultural values, outcome class) may explain our current mixed results, additional future research is needed to determine why and under what conditions these different pathways may become salient. Nevertheless, our study highlights the importance of considering earlier career events when it comes to predicting outcomes of job insecurity. In doing so, we add a life course perspective to the study of job insecurity and economic stressors, thus broadening our investigation of boundary conditions of job insecurity–outcome relationships beyond the current contemporaneous perspective.

## Data Availability

The data that support the findings of this study are openly available at https://osf.io/gqvyc/.

## Footnotes

1 We do not necessarily argue that perceiving job insecurity as less of a hindrance necessarily implies that it will be perceived as more of a challenge (i.e., a manageable and potentially expected part of one’s career), as challenge and hindrance job insecurity appraisals seem to be relatively independent of each other (Bazzoli et al., 2021).

## References

[CR1] Akkermans, J., Seibert, S. E., & Mol, S. T. (2018). Tales of the unexpected: Integrating career shocks in the contemporary careers literature. *South African Journal of Industrial Psychology,**44*, 1–10. 10.4102/sajip.v44i0.1503

[CR2] Akkermans, J., Rodrigues, R., Mol, S. T., Seibert, S. E., & Khapova, S. N. (2021). The role of career shocks in contemporary career development: Key challenges and ways forward. *Career Development International,**26*(4), 453–466. 10.1108/Cdi-07-2021-0172

[CR3] Amiri, S. (2022). Unemployment and suicide mortality, suicide attempts, and suicide ideation: A meta-analysis. *International Journal of Mental Health,**51*(4), 294–318. 10.1080/00207411.2020.1859347

[CR4] Armstrong-Stassen, M., & Ursel, N. D. (2009). Perceived organizational support, career satisfaction, and the retention of older workers. *Journal of Occupational and Organizational Psychology,**82*(1), 201–220. 10.1348/096317908x288838

[CR5] Atkinson, T., Liem, R., & Liem, J. H. (1986). The social costs of unemployment: Implications for social support. *Journal of Health and Social Behavior,**27*(4), 317–331. 10.2307/2136947 3559126

[CR6] Bandura, A. (1997). *Self-efficacy: The exercise of control*. Freeman.

[CR7] Barbaranelli, C., Fida, R., Paciello, M., & Tramontano, C. (2018). ‘Possunt, quia posse videntur’: They can because they think they can. Development and validation of the Work Self-Efficacy scale: Evidence from two studies. *Journal of Vocational Behavior,**106*, 249–269. 10.1016/j.jvb.2018.01.006

[CR8] Barling, J., & Weatherhead, J. G. (2016). Persistent exposure to poverty during childhood limits later leader emergence. *Journal of Applied Psychology,**101*(9), 1305–1318. 10.1037/apl0000129 27599090

[CR9] Batinic, B., Selenko, E., Stiglbauer, B., & Paul, K. I. (2010). Are workers in high-status jobs healthier than others? Assessing Jahoda’s latent benefits of employment in two working populations. *Work & Stress,**24*(1), 73–87. 10.1080/02678371003703859

[CR10] Bazzoli, A., & Probst, T. M. (2022). Taking stock and moving forward: A textual statistics approach to synthesizing four decades of job insecurity research. *Organizational Psychology Review,**12*(4), 507–544. 10.1177/20413866221112386

[CR11] Bazzoli, A., Probst, T. M., & Tomas, J. (2022). A latent profile analysis of precarity and its associated outcomes: The haves and the have-nots. *International Journal of Environmental Research and Public Health,**19*(13), 1–13. 10.3390/ijerph19137582 PMC9266012 35805236

[CR12] Bettac, E., & Probst, T. M. (2021). Work-family conflict, sleep, and health: A comparison of traditional and self-employed workers. International Journal of Manpower, 42(2), 240–259. 10.1108/IJM-02-2019-0106

[CR13] Bettac, E., Rice, S., & Probst, T. M. (2021). Job performance: Comparing differences among self- and organizationally-employed workers. Performance Improvement Quarterly, 34(1), 55–76. 10.1002/piq.21342

[CR14] Berntson, E., Näswall, K., & Sverke, M. (2010). The moderating role of employability in the association between job insecurity and exit, voice, loyalty and neglect. *Economic and Industrial Democracy,**31*(2), 215–230. 10.1177/0143831X09358374

[CR15] Bilendi. (n.d.). *ESOMAR – 37 questions to help buyers of online samples*. Author.

[CR16] Blau, G. J. (1985). The measurement and prediction of career commitment. *Journal of Occupational Psychology,**58*(4), 277–288. 10.1111/j.2044-8325.1985.tb00201.x

[CR17] Borg, I. (1992). Überlegungen und Untersuchungen zur Messung der subjektiven Unsicherheit der Arbeitsstelle [Reflections and studies on the measurement of subjective uncertainty of employment]. *Zeitschrift Für Arbeits- und Organisationspsychologie,**36*(3), 107–116.

[CR18] Borg, I., & Elizur, D. (1992). Job insecurity: Correlates, moderators and measurement. *International Journal of Manpower,**13*(2), 13–26. 10.1108/01437729210010210

[CR19] Bowling, N. A., & Hammond, G. D. (2008). A meta-analytic examination of the construct validity of the Michigan Organizational Assessment Questionnaire Job Satisfaction Subscale. *Journal of Vocational Behavior,**73*(1), 63–77. 10.1016/j.jvb.2008.01.004

[CR20] Brand, J. E. (2006). The effects of job displacement on job quality: Findings from the Wisconsin Longitudinal Study. *Research in Social Stratification and Mobility,**24*(3), 275–298. 10.1016/j.rssm.2006.03.001

[CR21] Cable, D. M., & Judge, T. A. (1996). Person-organization fit, job choice decisions, and organizational entry. *Organizational Behavior and Human Decision Processes,**67*(3), 294–311. 10.1006/obhd.1996.0081

[CR22] Callan, M. J., Shead, N. W., & Olson, J. M. (2011). Personal relative deprivation, delay discounting, and gambling. *Journal of Personality and Social Psychology,**101*(5), 955–973. 10.1037/a0024778 21875231

[CR23] Cammann, C., Fichman, M., Jenkins, G. D., & Klesh, J. (1983). Michigan Organizational Assessment Questionnaire. In S. E. Seashore, E. E. Lawler, P. H. Mirvis, & C. Cammann (Eds.), *Assessing organizational change: A guide to methods, measures, and practices* (pp. 71–138). John Wiley & Sons Inc.

[CR24] Cheng, G.H.-L., & Chan, D.K.-S. (2008). Who suffers more from job insecurity? A meta-analytic review. *Applied Psychology: An International Review,**57*(2), 272–303. 10.1111/j.1464-0597.2007.00312.x

[CR25] Cheng, T., Mauno, S., & Lee, C. (2014). The buffering effect of coping strategies in the relationship between job insecurity and employee well-being. *Economic and Industrial Democracy,**35*(1), 71–94. 10.1177/0143831X12463170

[CR26] Choi, S., Ko, K., Park, J. B., Lee, K. J., Lee, S., & Jeong, I. (2020). Combined effect of emotional labor and job insecurity on sleep disturbance among customer service workers. *Annals of Occupational and Environmental Medicine,**32*, e33. 10.35371/aoem.2020.32.e33 33072344 PMC7533295

[CR27] Clark, A. E., Georgellis, Y., & Sanfey, P. (2001). Scarring: The psychological impact of past unemployment. *Economica,**68*(270), 221–241. 10.1111/1468-0335.00243

[CR28] Cohen, J., Cohen, P., West, S. G., & Aiken, L. S. (2003). *Applied multiple regression/correlation analysis for the behavioral sciences* (3rd ed.). Lawrence Erlbaum.

[CR29] Crosby, F. (1976). A model of egoistical relative deprivation. *Psychological Review,**83*(2), 85–113. 10.1037/0033-295X.83.2.85

[CR30] Crosby, F. (1984). Relative deprivation in organizational settings. In B. M. Staw & L. L. Cummings (Eds.), *Research in Organizational Behavior* (pp. 51–93). JAI Press.

[CR31] Danckert, B. (2017). Facing unemployment: Personal and vicarious unemployment experiences generate favourable perceptions of unemployed people. *European Sociological Review,**33*(6), 779–790. 10.1093/esr/jcx076

[CR32] De Witte, H., Pienaar, J., & De Cuyper, N. (2016). Review of 30 years of longitudinal studies on the association between job insecurity and health and well-being: Is there causal evidence? *Australian Psychologist,**51*(1), 18–31. 10.1111/ap.12176

[CR33] Debus, M. E., Körner, B., Wang, M., & Kleinmann, M. (2023). Reacting to perceived over qualification: Uniting strain-based and self-regulatory adjustment reactions and the moderating role of formal work arrangements. *Journal of Business and Psychology*, *38*(2), 411–435. 10.1007/s10869-022-09870-8 PMC9852809 36694852

[CR34] Debus, M. E., & Unger, D. (2024). Disrupting the social and time vacuum: A systemic and lifespan perspective on job insecurity. *Applied Psychology: An International Review,**73*(4), 1994–2001. 10.1111/apps.12536

[CR35] Debus, M. E., Probst, T. M., König, C. J., & Kleinmann, M. (2012). Catch me if I fall! Enacted uncertainty avoidance and the social safety net as country-level moderators in the job insecurity–job attitudes link. *Journal of Applied Psychology,**97*(3), 690–698. 10.1037/a0027832 22448808

[CR36] Debus, M. E., König, C. J., & Kleinmann, M. (2014). The building blocks of job insecurity: The impact of environmental and person-related variables on job insecurity perceptions. *Journal of Occupational and Organizational Psychology,**87*(2), 329–351. 10.1111/joop.12049

[CR37] Debus, M. E., Kleinmann, M., König, C. J., & Winkler, S. (2020). Being tough versus tender: The impact of country-level and individual masculinity orientations as moderators of the relationship between job insecurity and job attitudes. *Applied Psychology: An International Review,**69*(3), 616–652. 10.1111/apps.12189

[CR38] Demerouti, E., & Rispens, S. (2014). Improving the image of student-recruited samples: A commentary. *Journal of Occupational and Organizational Psychology,**87*(1), 34–41. 10.1111/joop.12048

[CR39] Dweck, C. S., & Yeager, D. S. (2019). Mindsets: A view from two eras. *Perspectives on Psychological Science,**14*(3), 481–496. 10.1177/1745691618804166 30707853 PMC6594552

[CR40] Eaton, W. H. (1952). Hypotheses related to worker frustration. *The Journal of Social Psychology,**35*(1), 59–68. 10.1080/00224545.1952.9921830

[CR41] Edwards, J. R. (1991). Person-job fit: A conceptual integration, literature review, and methodological critique. In C. L. Cooper & I. T. Robertson (Eds.), *International Review of Industrial and Organizational Psychology* (pp. 283–357). Wiley.

[CR42] Eissa, G., & Lester, S. W. (2017). Supervisor role overload and frustration as antecedents of abusive supervision: The moderating role of supervisor personality. *Journal of Organizational Behavior,**38*(3), 307–326. 10.1002/job.2123

[CR43] Elder, G. H., Jr., & Shanahan, M. J. (2006). The life course and human development. In R. M. Lerner & W. Damon (Eds.), *Theoretical models of human development* (6th ed., pp. 665–715). Wiley.

[CR44] Elshaer, I. A., Ghanem, M., & Azazz, A. M. S. (2022). An unethical organizational behavior for the sake of the family: Perceived risk of job insecurity, family motivation and financial pressures. *International Journal of Environmental Research and Public Health,**19*(11), 6541. 10.3390/ijerph19116541 35682128 PMC9179977

[CR45] Erdogan, B., Tomás, I., Valls, V., & Gracia, F. J. (2018). Perceived overqualification, relative deprivation, and person centric outcomes: The moderating role of career centrality. *Journal of Vocational Behavior,**107*, 233–245. 10.1016/j.jvb.2018.05.003

[CR46] Fox, S., & Spector, P. E. (1999). A model of work frustration–Aggression. *Journal of Organizational Behavior,**20*(6), 915–931. 10.1002/(SICI)1099-1379(199911)20:6<915::AID-JOB918>3.0.CO;2-6

[CR47] Garcia-Lorenzo, L., Sell-Trujillo, L., & Donnelly, P. (2021). Responding to stigmatization: How to resist and overcome the stigma of unemployment. *Organization Studies,**43*(10), 1629–1650. 10.1177/01708406211053217

[CR48] Gedikli, C., Miraglia, M., Connolly, S., Bryan, M., & Watson, D. (2023). The relationship between unemployment and wellbeing: An updated meta-analysis of longitudinal evidence. *European Journal of Work and Organizational Psychology*, *32*(1). 10.1080/1359432x.2022.2106855

[CR49] Goldsmith, A. H., Veum, J. R., & Darity, W., Jr. (1996). The psychological impact of unemployment and joblessness. *The Journal of Socio-Economics,**25*(3), 333–358. 10.1016/S1053-5357(96)90009-8

[CR50] Goldsmith, A. H., Veum, J. R., & Darity, W., Jr. (1997). Unemployment, joblessness, psychological well-being and self-esteem: Theory and evidence. *The Journal of Socio-Economics,**26*(2), 133–158. 10.1016/S1053-5357(97)90030-5

[CR51] Goulet, L. R., & Singh, P. (2002). Career commitment: A reexamination and an extension. *Journal of Vocational Behavior,**61*(1), 73–91. 10.1006/jvbe.2001.1844

[CR52] Graham, B. A., & Sinclair, R. R. (2024). The chains of the past: A life course perspective on childhood adversity and organizational attitudes and behaviors. *Journal of Occupational Health Psychology,**29*(3), 155–173. 10.1037/ocp0000379 38913703

[CR53] Guarnaccia, C., Scrima, F., Civilleri, A., & Salerno, L. (2018). The role of occupational self-efficacy in mediating the effect of job insecurity on work engagement, satisfaction and general health. *Current Psychology,**37*(3), 488–497. 10.1007/s12144-016-9525-0

[CR54] Hall, J. A. (2019). How many hours does it take to make a friend? *Journal of Social and Personal Relationships,**36*(4), 1278–1296. 10.1177/0265407518761225

[CR55] Hanisch, K. A. (1992). The Job Descriptive Index revisited: Questions about the question mark. *Journal of Applied Psychology,**77*(3), 377–382. 10.1037/0021-9010.77.3.377

[CR56] Hayes, A. F. (2022). *Introduction to mediation, moderation, and conditional process analysis* (3rd ed.). Guilford Press.

[CR57] Hobfoll, S. E. (1989). Conservation of resources: A new attempt at conceptualizing stress. *American Psychologist,**44*(3), 513–524. 10.1037/0003-066X.44.3.513 2648906

[CR58] Hobfoll, S. E. (2001). The influence of culture, community, and the nested–self in the stress process: Advancing conservation of resources theory. *Applied Psychology: An International Review,**50*(3), 337–421. 10.1111/1464-0597.00062

[CR59] Hobfoll, S. E., Halbesleben, J., Neveu, J. P., & Westman, M. (2018). Conservation of resources in the organizational context: The reality of resources and their consequences. *Annual Review of Organizational Psychology and Organizational Behavior,**5*, 103–128. 10.1146/annurev-orgpsych-032117-104640

[CR60] Hofer, A., Spurk, D., & Hirschi, A. (2021). When and why do negative organization-related career shocks impair career optimism? A conditional indirect effect model. *Career Development International,**26*(4), 467–494. 10.1108/Cdi-12-2018-0299

[CR61] Hofstede, G. (1998). Masculinity/femininity as a dimension of culture. In G. Hofstede (Ed.), *Masculinity and femininity: The taboo dimension of national cultures* (pp. 3–28). Sage.

[CR62] Hofstede, G. (2001). *Culture’s consequences* (2nd ed.). Sage.

[CR63] Holman, E. A., & Silver, R. C. (1998). Getting “stuck” in the past: Temporal orientation and coping with trauma. *Journal of Personality and Social Psychology,**74*(5), 1146–1163. 10.1037/0022-3514.74.5.1146 9599436

[CR64] Howard, M. C., & Hoffman, M. E. (2018). Variable-centered, person-centered, and person-specific approaches: Where theory meets the method. *Organizational Research Methods,**21*(4), 846–876. 10.1177/1094428117744

[CR65] Jahoda, M. (1981). Work, employment, and unemployment: Values, theories, and approaches in social research. *American Psychologist,**36*(2), 184–191. 10.1037/0003-066X.36.2.184

[CR66] Jahoda, M. (1982). *Employment and unemployment: A social-psychological analysis*. Cambridge University Press.

[CR67] Jiang, L. X., & Lavaysse, L. M. (2018). Cognitive and affective job insecurity: A meta-analysis and a primary study. *Journal of Management,**44*(6), 2307–2342. 10.1177/0149206318773853

[CR68] Jiang, L. X., & Probst, T. M. (2014). Organizational communication: A buffer in times of job insecurity? *Economic and Industrial Democracy,**35*(3), 557–579. 10.1177/0143831x13489356

[CR69] Jiang, L. (2025). An integrative review of moderators attenuating or exacerbating negative outcomes of job insecurity. *Academy of Management Annals.* Advance online publication. 10.5465/annals.2024.0040

[CR70] Kammeyer-Mueller, J. D., Judge, T. A., & Piccolo, R. F. (2008). Self-esteem and extrinsic career success: Test of a dynamic model. *Applied Psychology: An International Review,**57*(2), 204–224. 10.1111/j.1464-0597.2007.00300.x

[CR71] Kecklund, G., & Åkerstedt, T. (1992). The pattern of slow wave activity in spontaneously occurring long sleep. *Journal of Sleep Research,**1*(1), 30–34. 10.1111/j.1365-2869.1992.tb00005.x 10607022

[CR72] Keim, A. C., Landis, R. S., Pierce, C. A., & Earnest, D. R. (2014). Why do employees worry about their jobs? A meta-analytic review of predictors of job insecurity. *Journal of Occupational Health Psychology,**19*(3), 269–290. 10.1037/A0036743 24796228

[CR73] Kinnunen, U., Feldt, T., & Mauno, S. (2003). Job insecurity and self-esteem: Evidence from cross-lagged relations in a 1-year longitudinal sample. *Personality and Individual Differences,**35*(3), 617–632. 10.1016/S0191-8869(02)00223-4

[CR74] Klopack, E. T., Wickrama, K. A. S., & Simons, R. L. (2022). Life course trajectories of chronic financial strain and acute stress reactivity: Steeling in response to recovery from strain. *Stress & Health,**38*(2), 277–289. 10.1002/smi.3087 34379875 PMC9190465

[CR75] Köhler, T., & Cortina, J. M. (2021). Play it again, Sam! An analysis of constructive replication in the organizational sciences. *Journal of Management,**47*(2), 488–518. 10.1177/0149206319843985

[CR76] Kok, A. A. L., Twisk, J. W. R., Blom, F., Beekman, A. T. F., & Huisman, M. (2021). Steeling or sensitizing? A longitudinal examination of how ongoing accumulation of negative life events affects depressive symptoms in older adults. *The Journals of Gerontology,**76*(10), 2041–2053. 10.1093/geronb/gbab114 34171092 PMC8599083

[CR77] Lazarus, R. S. (1991). *Emotion and adaptation*. Oxford University Press.

[CR78] Lazarus, R. S. (1999). *Stress and emotion: A new synthesis*. Springer.

[CR79] Lazarus, R. S. (2001). *Emotion and adaptation*. Springer.

[CR80] Lazauskaite-Zabielske, J., Urbanaviciute, I., Vander Elst, T., & De Witte, H. (2019). Explaining the link between qualitative job insecurity and attitudes: The role of perceived overall justice. *Baltic Journal of Management,**14*(2), 330–344. 10.1108/BJM-08-2018-0293

[CR81] Lee, C., Huang, G.-H., & Ashford, S. J. (2018). Job insecurity and the changing workplace: Recent developments and the future trends in job insecurity research. *Annual Review of Organizational Psychology and Organizational Behavior,**55*(1), 335–359. 10.1146/annurev-orgpsych-032117-104651

[CR82] Lee, H. J., Tomas, J., Probst, T. M., & Lindgren, R. J. (2024). Production pressure, cognitive failures, and injuries under an insecure job climate. *International Journal of Stress Management*, *31*(2), 174–183. 10.1037/str0000315

[CR83] Lin, W. P., Shao, Y. D., Li, G. Q., Guo, Y. R., & Zhan, X. J. (2021). The psychological implications of COVID-19 on employee job insecurity and its consequences: The mitigating role of organization adaptive practices. *Journal of Applied Psychology,**106*(3), 317–329. 10.1037/apl0000896 33871269

[CR84] Liu, D., Zhang, Z., & Wang, M. (2012). Mono-level and multilevel mediated moderation and moderated mediation: Theorization and test. In X. Chen, A. S. Tsui, & J. L. Farh (Eds.), *Empirical methods in organization and management research* (pp. 553–587). Peking University Press.

[CR85] López Bohle, S. A., Chambel, M. J., & Diaz-Valdes Iriarte, A. (2021). Job insecurity, procedural justice and downsizing survivor affects. *International Journal of Human Resource Management,**32*(3), 596–615. 10.1080/09585192.2018.1482939

[CR86] Lu, C., Wang, H., Lu, J., Du, D. Y., & Bakker, A. (2014). Does work engagement increase person–job fit? The role of job crafting and job insecurity. *Journal of Vocational Behavior,**84*(2), 142–152. 10.1016/j.jvb.2013.12.004

[CR87] Lucas, R. E., Clark, A. E., Georgellis, Y., & Diener, E. (2004). Unemployment alters the set point for life satisfaction. *Psychological Science,**15*(1), 8–13. 10.1111/j.0963-7214.2004.01501002.x 14717825

[CR88] Luhmann, M., & Eid, M. (2009). Does it really feel the same? Changes in life satisfaction following repeated life events. *Journal of Personality and Social Psychology,**97*(2), 363–381. 10.1037/A0015809 19634981

[CR89] Luksyte, A., Bauer, T., Debus, M. E., Erdogan, B., & Wu, C. H. (2022). Perceived overqualification and collectivism orientation: Implications for work and nonwork outcomes. *Journal of Management,**48*(2), 319–349. 10.1177/0149206320948602

[CR90] Luo, J., Zhang, B., & Roberts, B. W. (2021). Sensitization or inoculation: Investigating the effects of early adversity on personality traits and stress experiences in adulthood. *Plos One,**16*(4), e0248822. 10.1371/journal.pone.0248822 33793582 PMC8016298

[CR91] McKee-Ryan, F. M., Song, Z. L., Wanberg, C. R., & Kinicki, A. J. (2005). Psychological and physical well-being during unemployment: A meta-analytic study. *Journal of Applied Psychology,**90*(1), 53–76. 10.1037/0021-9010.90.1.53 15641890

[CR92] Meehl, P. E. (1990). Why summaries of research on psychological theories are often uninterpretable. *Psychological Reports,**66*(1), 195–244. 10.2466/pr0.1990.66.1

[CR93] Meichenbaum, D. H., & Deffenbacher, J. L. (1988). Stress inoculation training. *The Counseling Psychologist,**16*(1), 69–90. 10.1177/0011000088161005

[CR94] Mishra, S., & Novakowski, D. (2016). Personal relative deprivation and risk: An examination of individual differences in personality, attitudes, and behavioral outcomes. *Personality and Individual Differences,**90*, 22–26. 10.1016/j.paid.2015.10.031

[CR95] Molleman, E., Pruyn, J., & Van Knippenberg, A. (1986). Social comparison processes among cancer patients. *British Journal of Social Psychology*, *25*(1). 10.1111/j.2044-8309.1986.tb00695.x 3947800

[CR96] Muthén, L. K., & Muthén, B. O. (1998–2017). *Mplus user’s guide* (8th ed.). Muthén & Muthén.

[CR97] Neal, A., Griffin, M. A., & Hart, P. M. (2000). The impact of organizational climate on safety climate and individual behavior. *Safety Science,**34*(1–3), 99–109. 10.1016/S0925-7535(00)00008-4

[CR98] Nolte-Troha, C., Roser, P., Henkel, D., Scherbaum, N., Koller, G., & Franke, A. G. (2023). Unemployment and substance use: An updated review of studies from north America and Europe. *Healthcare,**11*(8), 1182. 10.3390/healthcare11081182 37108016 PMC10137824

[CR99] O’Boyle, E. H., Jr., Banks, G. C., & Gonzalez-Mulé, E. (2017). The chrysalis effect: How ugly initial results metamorphosize into beautiful articles. *Journal of Management,**43*(2), 376–399. 10.1177/0149206314527133

[CR100] Oki, A. (2013). *Too overqualified to care: The effect of cynicism on overqualification and commitment* [Doctoral dissertation, University of Houston].

[CR101] Oswald, M. E., & Grosjean, S. (2004). Confirmation bias. In R. F. Pohl (Ed.), *Cognitive illusions: A handbook on fallacies and biases in thinking, judgement and memory* (pp. 79–96). Psychology Press.

[CR102] Patton, W., & Donohue, R. (1998). Coping with long-term unemployment. *Journal of Community & Applied Social Psychology,**8*(5), 331–343. 10.1002/(SICI)1099-1298(1998090)8:5<331::AID-CASP456>3.0.CO;2-6

[CR103] Paul, K. I., & Moser, K. (2009). Unemployment impairs mental health: Meta-analyses. *Journal of Vocational Behavior,**74*(3), 264–282. 10.1016/j.jvb.2009.01.001

[CR104] Paul, K. I., Scholl, J., Moser, K., Zechmann, A., & Batinic, B. (2023). Employment status, psychological needs, and mental health: Meta-analytic findings concerning the latent deprivation model. *Frontiers in Psychology,**14*, 1017358. 10.3389/fpsyg.2023.1017358 36935981 PMC10017486

[CR105] Peer, E., Vosgerau, J., & Acquisti, A. (2014). Reputation as a sufficient condition for data quality on Amazon Mechanical Turk. *Behavior Research Methods,**46*(4), 1023–1031. 10.3758/s13428-013-0434-y 24356996

[CR106] Peters, L. H., O’Connor, E., & Rudolf, C. J. (1980). The behavioral and affective consequences of performance-relevant situational variables. *Organizational Behavior and Human Performance,**25*(1), 79–96. 10.1016/0030-5073(80)90026-4

[CR107] Petitta, L., Probst, T. M., Ghezzi, V., & Barbaranelli, C. (2019). Cognitive failures in response to emotional contagion: Their effects on workplace accidents. *Accident Analysis & Prevention*, *119*, 165–173. 10.1016/j.aap.2019.01.018 30763814

[CR108] Petitta, L., Probst, T. M., Barbaranelli, C., & Ghezzi, V. (2020). Economic stress, emotional contagion and safety outcomes: A cross-country study. *Work*, *66*(2), 421–435. 10.3233/WOR-203182 32568157

[CR109] Petitta, L., Probst, T. M., Ghezzi, V., & Barbaranelli, C. (2021). Emotional contagion as a trigger for moral disengagement: Their effects on workplace injuries. *Safety Science*, *140*, 105317. 10.1016/j.ssci.2021.105317

[CR110] Picchio, M., & Ubaldi, M. (2024). Unemployment and health: A meta-analysis. *Journal of Economic Surveys,**38*(4), 1437–1472. 10.1111/joes.12588

[CR111] Piccoli, B., & De Witte, H. (2015). Job insecurity and emotional exhaustion: Testing psychological contract breach versus distributive injustice as indicators of lack of reciprocity. *Work & Stress,**29*(3), 246–263. 10.1080/02678373.2015.1075624

[CR112] Platt, J. R. (1964). Strong inference: Certain systematic methods of scientific thinking may produce much more rapid progress than others. *Science,**146*(3642), 347–353. 10.1126/science.146.3642.34 17739513

[CR113] Porath, C. L., & Pearson, C. M. (2012). Emotional and behavioral responses to workplace incivility and the impact of hierarchical status. *Journal of Applied Social Psychology,**42*(1), 326–357. 10.1111/j.1559-1816.2012.01020.x

[CR114] Powdthavee, N. (2012). Jobless, friendless and broke: What happens to different areas of life before and after unemployment. *Economica,**79*(315), 557–575. 10.1111/j.1468-0335.2011.00905.x

[CR115] Preacher, K. J., Rucker, D. D., & Hayes, A. F. (2007). Addressing moderated mediation hypotheses: Theory, methods, and prescriptions. *Multivariate Behavioral Research,**42*(1), 185–227. 10.1080/00273170701341316 26821081

[CR116] Probst, T. M. (2003). Development and validation of the Job Security Index and the Job Security Satisfaction scale: A classical test theory and IRT approach. *Journal of Occupational and Organizational Psychology,**76*(4), 451–467. 10.1348/096317903322591587

[CR117] Probst, T. M., Jiang, L., & Benson, W. L. (2018). Job insecurity and anticipated job loss: A primer and exploration of possible interventions. In U. C. Klehe & E. A. J. van Hooft (Eds.), *The Oxford handbook of job loss and job search* (pp. 31–53). Oxford University Press.

[CR118] Probst, T. M., Chizh, A., Hu, S. M., Jiang, L. X., & Austin, C. (2020a). Explaining the relationship between job insecurity and creativity: A test of cognitive and affective mediators. *Career Development International,**25*(3), 247–270. 10.1108/Cdi-04-2018-0118

[CR119] Probst, T. M., Lee, H. J., & Bazzoli, A. (2020b). Economic stressors and the enactment of CDC-recommended COVID-19 prevention behaviors: The impact of state-level context. *Journal of Applied Psychology,**105*(12), 1397–1407. 10.1037/apl0000797 33271028

[CR120] Probst, T. M., Petitta, L., Barbaranelli, C., & Austin, C. (2020c). Safety-related moral disengagement in response to job insecurity: Counter intuitive effects of perceived organizational and supervisor support. *Journal of Business Ethics*, *162*(2), 343–358. 10.1007/s10551-018-4002-3

[CR121] Reio, T. G. (2011). Supervisor and coworker incivility: Testing the work frustration-aggression model. *Advances in Developing Human Resources,**13*(1), 54–68. 10.1177/1523422311410648

[CR122] Reisel, W. D., Probst, T. M., Chia, S.-L., Maloles, C. M., III., & König, C. J. (2010). The effects of job insecurity on job satisfaction, organizational citizenship behavior, deviant behavior, and negative emotions of employees. *International Studies of Management and Organization,**40*(1), 74–91. 10.2753/IMO0020-8825400105

[CR123] Rousseau, D. M. (1995). *Psychological contracts in organizations: Understanding written and unwritten agreements*. Sage.

[CR124] Saks, A. M., & Ashforth, B. E. (1997). A longitudinal investigation of the relationships between job information sources, applicant perceptions of fit, and work outcomes. *Personnel Psychology,**50*(2), 395–426. 10.1111/j.1744-6570.1997.tb00913.x

[CR125] Seery, M. D., Leo, R. J., Holman, E. A., & Cohen Silver, R. (2010). Lifetime exposure to adversity predicts functional impairment and healthcare utilization among individuals with chronic back pain. *Pain,**150*(3), 507–515. 10.1016/j.pain.2010.06.007 20594645

[CR126] Seery, M. D., Leo, R. J., Lupien, S. P., Kondrak, C. L., & Almonte, J. L. (2013). An upside to adversity? Moderate cumulative lifetime adversity is associated with resilient responses in the face of controlled stressors. *Psychological Science,**24*(7), 1181–1189. 10.1177/0956797612469210 23673992

[CR127] Segura, S., & González-Romá, V. (2003). How do respondents construe ambiguous response formats of affect items? *Journal of Personality and Social Psychology,**85*(5), 956–968. 10.1037/0022-3514.85.5.956 14599257

[CR128] Seibert, S. E., Kraimer, M. L., Holtom, B. C., & Pierotti, A. J. (2013). Even the best laid plans sometimes go askew: Career self-management processes, career shocks, and the decision to pursue graduate education. *Journal of Applied Psychology,**98*(1), 169–182. 10.1037/a0030882 23181345

[CR129] Selenko, E., & Batinic, B. (2013). Job insecurity and the benefits of work. *European Journal of Work and Organizational Psychology,**22*(6), 725–736. 10.1080/1359432x.2012.703376

[CR130] Selenko, E., Batinic, B., & Paul, K. (2011). Does latent deprivation lead to psychological distress? Investigating Jahoda’s model in a four-wave study. *Journal of Occupational and Organizational Psychology,**84*(4), 723–740. 10.1348/096317910x519360

[CR131] Selig, J. P., & Preacher, K. J. (2008). *Monte Carlo method for assessing mediation: An interactive tool for creating confidence intervals for indirect effects*. http://quantpsy.org/medmc/medmc.htm

[CR132] Shipp, A. J., & Jansen, K. J. (2011). Reinterpreting time in fit theory: Crafting and recrafting narratives of fit in medias res. *Academy of Management Review,**36*(1), 76–101. 10.5465/amr.2009.0077

[CR133] Shoss, M. K. (2017). Job insecurity: An integrative review and agenda for future research. *Journal of Management,**43*(6), 1911–1939. 10.1177/0149206317691574

[CR134] Shoss, M. K., Jiang, L., & Probst, T. M. (2018). Bending without breaking: A two-study examination of employee resilience in the face of job insecurity. *Journal of Occupational Health Psychology,**23*(1), 112–126. 10.1037/ocp0000060 27786505

[CR135] Sinclair, R. R., Probst, T. M., Paige Watson, G., & Bazzoli, A. (2021). Caught between Scylla and Charybdis: How economic stressors and occupational risk factors influence workers’ occupational health reactions to COVID-19. *Applied Psychology: An International Review,**70*(1), 85–119. 10.1111/apps.12301 PMC7753445 33362328

[CR136] Sinclair, R. R., Graham, B. A., & Probst, T. M. (2024). Economic stress and occupational health. *Annual Review of Organizational Psychology and Organizational Behavior,**11*, 423–451. 10.1146/annurev-orgpsych-091922-020639

[CR137] Smid, G. E., van der Velden, P. G., Lensvelt-Mulders, G. J. L. M., Knipscheer, J. W., Gersons, B. P. R., & Kleber, R. J. (2012). Stress sensitization following a disaster: A prospective study. *Psychological Medicine,**42*(8), 1675–1686. 10.1017/S0033291711002765 22126800

[CR138] Smith, H. J., Pettigrew, T. F., Pippin, G. M., & Bialosiewicz, S. (2011). Relative deprivation: A theoretical and meta-analytic review. *Personality and Social Psychology Review,**16*(3), 203–232. 10.1177/1088868311430825 22194251

[CR139] Spector, P. E. (1978). Organizational frustration: A model and review of the literature. *Personnel Psychology,**31*(4), 815–829. 10.1111/j.1744-6570.1978.tb02125.x

[CR140] Staufenbiel, T., & Hartz, C. (2000). Organizational citizenship behavior: Entwicklung und erste Validierung eines Messinstruments [Organizational citizenship behavior: Development and validation of a measurement instrument]. *Diagnostica,**46*(2), 73–83. 10.1026/0012-1924.46.2.73

[CR141] Staufenbiel, T., & König, C. J. (2010). A model for the effects of job insecurity on performance, turnover intention, and absenteeism. *Journal of Occupational and Organizational Psychology,**83*(1), 101–117. 10.1348/096317908X401912

[CR142] Sverke, M., Hellgren, J., & Näswall, K. (2002). No security: A meta-analysis and review of job insecurity and its consequences. *Journal of Occupational Health Psychology,**7*(3), 242–264. 10.1037/1076-8998.7.3.242 12148956

[CR143] Sverke, M., Låstad, L., Hellgren, J., Richter, A., & Näswall, K. (2019). A meta-analysis of job insecurity and employee performance: Testing temporal aspects, rating source, welfare regime, and union density as moderators. *International Journal of Environmental Research and Public Health,**16*(14), 2536. 10.3390/ijerph16142536 31315198 PMC6678210

[CR144] Talentlms. (2024). *What employees want in 2024: Top 10 things they look for*. https://www.talentlms.com/blog/what-employees-want/

[CR145] Taylor, S. E., & Lobel, M. (1989). Social comparison activity under threat: Downward evaluation and upward contacts. *Psychological Review,**96*(4), 569–575. 10.1037/0033-295X.96.4.569 2678204

[CR146] Thoits, P. A. (2010). Stress and health: Major findings and policy implications. *Journal of Health and Social Behavior,**51*(1_suppl), S41–S53. 10.1177/0022146510383499 20943582

[CR147] Van Hootegem, A., Sverke, M., & De Witte, H. (2021). Does occupational self-efficacy mediate the relationships between job insecurity and work-related learning? A latent growth modelling approach. *Work & Stress,**36*(3), 229–250. 10.1080/02678373.2021.1891585

[CR148] Van Katwyk, P. T., Fox, S., Spector, P. E., & Kelloway, E. K. (2000). Using the Job-Related Affective Well-Being Scale (JAWS) to investigate affective responses to work stressors. *Journal of Occupational Health Psychology,**5*(2), 219–230. 10.1037/1076-8998.5.2.219 10784286

[CR149] van Woerkom, M., Bakker, A. B., & Nishii, L. H. (2016). Accumulative job demands and support for strength use: Fine-tuning the job demands-resources model using conservation of resources theory. *Journal of Applied Psychology,**101*(1), 141–150. 10.1037/apl0000033 26121090

[CR150] Vander Elst, T., Baillien, E., De Cuyper, N., & De Witte, H. (2010). The role of organizational communication and participation in reducing job insecurity and its negative association with work-related well-being. *Economic and Industrial Democracy,**31*, 249–264. 10.1177/0143831X09358372

[CR151] Vander Elst, T., Van den Broeck, A., De Witte, H., & De Cuyper, N. (2012). The mediating role of frustration of psychological needs in the relationship between job insecurity and work-related well-being. *Work & Stress,**26*(3), 252–271. 10.1080/02678373.2012.703900

[CR152] Vesalainen, J., & Vuori, J. (1999). Job-seeking, adaptation and re-employment experiences of the unemployed: A 3-year follow-up. *Journal of Community & Applied Social Psychology,**9*(5), 383–394. 10.1002/(Sici)1099-1298(199909/10)9:5<383::Aid-Casp530>3.0.Co;2-0

[CR153] Voss, K., Markiewicz, D., & Doyle, A. B. (1999). Friendship, marriage and self-esteem. *Journal of Social and Personal Relationships,**16*(1), 103–122. 10.1177/0265407599161006

[CR154] Wallace, J. C., & Chen, G. (2005). Development and validation of a work-specific measure of cognitive failure: Implications for occupational safety. *Journal of Occupational and Organizational Psychology,**78*(4), 615–632. 10.1348/096317905X37442

[CR155] Wanberg, C. R. (1997). Antecedents and outcomes of coping behaviors among unemployed and reemployed individuals. *Journal of Applied Pschology,**82*(5), 731–744. 10.1037/0021-9010.82.5.731 9337607

[CR156] Wanberg, C. R. (2012). The individual experience of unemployment. *Annual Review of Psychology,**63*, 369–396. 10.1146/annurev-psych-120710-100500 21721936

[CR157] Waters, L. (2007). Experiential differences between voluntary and involuntary job redundancy on depression, job-search activity, affective employee outcomes and re-employment quality. *Journal of Occupational and Organizational Psychology,**80*(2), 279–299. 10.1348/096317906x104004

[CR158] Wickham, S., Shyrane, N., Lyons, M., Dickins, T., & Bentall, R. (2014). Why does relative deprivation affect mental health? The role of justice, trust and social rank in psychological wellbeing and paranoid ideation. *Journal of Public Mental Health,**13*(2), 114–126. 10.1108/JPMH-06-2013-0049

[CR159] Wigert, B. (2022). *The top 6 things employees want in their next job*. Gallup. https://www.gallup.com/workplace/389807/top-things-employees-next-job.aspx

[CR160] Wilke, R. A. (2005). New estimates of the duration and risk of unemployment for West-Germany. *Journal of Contextual Economics - Schmoller’s Jahrbuch,**125*(2), 207–237.

[CR161] Williams, L. J., & Anderson, S. E. (1991). Job satisfaction and organizational commitment as predictors of organizational citizenship and in-role behaviors. *Journal of Management,**17*(3), 601–617. 10.1177/014920639101700305

[CR162] Woo, S. E., Hofmans, J., Wille, B., & Tay, L. (2024). Person-centered modeling: Techniques for studying associations between people rather than variables. *Annual Review of Organizational Psychology and Organizational Behavior,**11*, 453–480. 10.1146/annurev-orgpsych-110721-045646

[CR163] Zacher, H., & Froidevaux, A. (2021). Life stage, lifespan, and life course perspectives on vocational behavior and development: A theoretical framework, review, and research agenda. *Journal of Vocational Behavior,**126*, 103476. 10.1016/j.jvb.2020.103476

[CR164] Zikic, J., & Klehe, U. C. (2006). Job loss as a blessing in disguise: The role of career exploration and career planning in predicting reemployment quality. *Journal of Vocational Behavior,**69*, 391–409. 10.1016/j.jvb.2006.05.007

